# Splicing analysis for exonic and intronic mismatch repair gene variants associated with Lynch syndrome confirms high concordance between minigene assays and patient RNA analyses

**DOI:** 10.1002/mgg3.145

**Published:** 2015-04-23

**Authors:** Heleen M van der Klift, Anne M L Jansen, Niki van der Steenstraten, Elsa C Bik, Carli M J Tops, Peter Devilee, Juul T Wijnen

**Affiliations:** 1 Department of Human Genetics, Leiden University Medical CenterLeiden, The Netherlands; 2 Department of Clinical Genetics, Leiden University Medical CenterLeiden, The Netherlands; 3 Department of Pathology, Leiden University Medical CenterLeiden, The Netherlands

**Keywords:** Aberrant splicing, Lynch syndrome, minigene assay, mismatch repair genes, patient RNA analysis, variant classification

## Abstract

A subset of DNA variants causes genetic disease through aberrant splicing. Experimental splicing assays, either RT-PCR analyses of patient RNA or functional splicing reporter minigene assays, are required to evaluate the molecular nature of the splice defect. Here, we present minigene assays performed for 17 variants in the consensus splice site regions, 14 exonic variants outside these regions, and two deep intronic variants, all in the DNA mismatch-repair (MMR) genes *MLH1*, *MSH2*, *MSH6*, and *PMS2*, associated with Lynch syndrome. We also included two deep intronic variants in *APC* and *PKD2*. For one variant (*MLH1* c.122A>G), our minigene assay and patient RNA analysis could not confirm the previously reported aberrant splicing. The aim of our study was to further investigate the concordance between minigene splicing assays and patient RNA analyses. For 30 variants results from patient RNA analyses were available, either performed by our laboratory or presented in literature. Some variants were deliberately included in this study because they resulted in multiple aberrant transcripts in patient RNA analysis, or caused a splice effect other than the prevalent exon skip. While both methods were completely concordant in the assessment of splice effects, four variants exhibited major differences in aberrant splice patterns. Based on the present and earlier studies, together showing an almost 100% concordance of minigene assays with patient RNA analyses, we discuss the weight given to minigene splicing assays in the current criteria proposed by InSiGHT for clinical classification of MMR variants.

## Introduction

An estimated 15–60% of pathogenic mutations cause genetic disease through disruption of constitutional pre-mRNA splicing (Wang and Cooper 2007). Variants in the consensus donor (5′) and acceptor (3′) splice site regions – defined as the last three exonic to the first six intronic bases, and the last 12 intronic to the first two exonic bases, respectively (Cartegni et al. 2002) – can abolish or diminish the strength of canonical splice sites. Additionally, exonic and intronic variants outside these regions may also affect splicing, either by creation of new splice sites, activation of existing cryptic splice sites, or by altering splice regulatory elements (SREs). Although all types of variants, including nonsense mutations and frame-shifting insertions and deletions, can disturb constitutional splicing, in molecular diagnostic practice often only variants of uncertain significance (VUS) are investigated for aberrant splicing. VUS, such as intronic variants, missense and silent variants, cannot be considered pathogenic without further supportive evidence. A combination of in silico prediction tools and “wet-lab” experiments such as RT-PCR analysis of patient-derived RNA or a functional splicing reporter minigene assay can be used to assess the effect of these variants on mRNA splicing (Baralle et al. 2009).

Although patient RNA is usually preferred for splicing analysis, several issues such as availability, degradation of aberrant transcripts through nonsense-mediated mRNA decay (NMD), and the confounding presence of normal and alternative transcripts from the wild-type allele in heterozygous patients can hamper straightforward analysis of aberrant splicing from the variant allele. As an alternative, minigene assays can be performed using genomic DNA from the patient or control DNA in which the variant is created by site-directed mutagenesis (SDM). It has been shown previously that minigene assays display high sensitivity and specificity in the assessment of aberrant splicing caused by genetic sequence variants (Tournier et al. 2008). However, occasional differences in splice patterns are observed between minigene and patient RNA analysis (Bonnet et al. 2008; Acedo et al. 2012; Steffensen et al. 2014).

The aim of our study was to further investigate the concordance between minigene assays and patient RNA analyses, focusing especially on differences and similarities in splice patterns produced by the variant allele in both tests. For this purpose, we also included variants that resulted in multiple aberrant transcripts in patient RNA analysis, or that caused another splice effect than the prevalent exon skip. We tested 33 variants found in the mismatch repair (MMR) genes *MLH1* (MIM*120436), *PMS2* (MIM*600259), *MSH2* (MIM*609309), and *MSH6* (MIM*600678), for aberrant splicing using the previously described pCAS2 minigene vector (Tournier et al. 2008; Gaildrat et al. 2010). Heterozygous mutations in the MMR genes cause Lynch syndrome (OMIM #120435), an autosomal dominant predisposition for colorectal, endometrial, and other cancers (Lynch and de la Chapelle 2003), whereas biallelic mutations lead to constitutional MMR deficiency syndrome (CMMRD; OMIM #276300) in which various types of malignancy occur early in life (Wimmer and Etzler 2008). We performed minigene assays for 17 MMR gene variants in the consensus splice site regions, 14 exonic variants outside these regions, and two deep intronic variants. We expanded this last category by including a *PKD2* (MIM*173910) and an *APC* (MIM*611731) deep intronic variant reported in literature (Rossetti et al. 2012; Spier et al. 2012). For 30 variants, patient RNA analysis data were available, either from our laboratory or reported by others. We found 100% concordance between patient RNA analysis and the minigene assay in the assessment of variants in terms of causing a splice effect or not, thereby confirming the high concordances found in previous studies. For four variants we observed major differences in aberrant splice patterns between the two tests but this did not influence the assessment of pathogenicity of the variant. On the basis of these and previous results, we suggest that minigene assay results deserve a more prominent position amongst the criteria recently presented by the International Society for Gastrointestinal Hereditary Tumours (InSiGHT) for clinical classification of MMR gene variants (Thompson et al. 2014).

## Material and Methods

### Selection of variants for minigene assays

Twelve *MLH1*, sixteen *PMS2*, four *MSH2*, and one *MSH6* sequence variant were tested for their effects on splicing with minigene assays (Table1). The variants were mainly detected in genomic DNA of Lynch syndrome patients undergoing genetic testing at our molecular diagnostic laboratory (LDGA, Leiden University Medical Center, Leiden, The Netherlands). Three *PMS2* variants, two of which produced multiple aberrant transcripts in patient RNA, were chosen from literature (*PMS2* c.989-1G>T, Sjursen et al. 2009; *PMS2* c.538-3C>G and c.989-2A>G, Borras et al. 2013). Seventeen variants were located in the consensus splice site regions, including nine at the canonical −1, −2, +1, +2 positions. Fourteen variants were situated deeper in the exon outside the consensus splice site regions. Furthermore, two deep intronic *MSH2* variants were included, one found by targeted next-generation sequencing in our laboratory, whereas the other was reported in literature (*MSH2* c.212-478T>G; Clendenning et al. 2011). This last *MSH2* variant is, to the best of our knowledge, the only deep intronic MMR gene variant yet found that causes aberrant splicing through pseudoexon inclusion. We expanded the deep intronic variant category by including a deep intronic *APC* variant (c.532-941G>A; Spier et al. 2012) and a deep intronic *PKD2* variant (c.1094+507A>G; Rossetti et al. 2012). Variants were classified in four categories according to their position relative to the consensus splice site regions:

1. at the canonical −1, −2, +1, +2 position (nine variants)
2. in the consensus splice site regions excluding the canonical positions (eight variants)
3. exonic outside the consensus splice site regions (14 variants)
4. deep intronic (four variants)

**Table 1:** Splice patterns for 20 wild-type and 35 variant alleles, produced by pCAS2 minigenes transfected in HEK293 and/or HeLa cells^1^.

Exon(s) included in minigene amplicon^2^	Splice pattern of WILDTYPE allele	Variant^3^	Variant category^4^	Position in exon to acceptor (+) or donor (−) splice site	Splice pattern of VARIANT allele
MLH1 exon 2	FL + minor Δ2p_5nts	c.122A>G (p.Asp41Gly)	3	(+6)	Same as WT; minor increase of Δ2p_5nts
MLH1 exon 3	FL + minor Δ3q_5nts (Hela nt)	c.277A>G (p.Ser93Gly)	3	(−30)	Same as WT (Hela nt)
MLH1 exon 6	FL + minor Δ6	c.543C>G (p.=)	2	(−3)	Δ6 (Hela nt)
		c.545G>A (p.Arg182Lys)	2	(−1)	Two transcripts: Δ6 (major) + Δ6q_4nts (minor)
MLH1 exon 10	FL + minor Δ10	c.791-1G>C	1	na	Δ10
		c.793C>A (p.Arg265Ser)	3	(+3)	Δ10
		c.793C>T (p.Arg265Cys)	3	(+3)	Partial splice effect: Δ10 (major) + FL (minor)
		c.882C>T (p.=)	2	(−3)	Δ10
		c.883A>G (p.Ser295Gly)	2	(−2)	Δ10
MLH1 exon 14	FL (Hela nt)	c.1633A>G (p.Thr545Ala)	3	(−35)	Same as WT (Hela nt)
		c.1667+1del	1	na	▾14q_87nts (=c.1667+2_1667+88) (Hela nt)
MLH1 exon 17+18	FL (Hela nt)	c.2103G>A (p.=)	2	(Exon 18; −1)	Three transcripts: Δ18 (major) + Δ[17+18] (very minor) + FL (very minor) (Hela nt)
PMS2 exon 2	FL + minor Δ2p_5nts	c.139C>T (p.=)	3	(−25)	Same as WT
		c.163+2T>C	1	na	Δ2
PMS2 exon 3+4	Three transcripts: HEK293: FL (major) + Δ4q_53nts (minor) + Δ4 (major); HeLa: same transcripts but only Δ4 with major expression	c.180C>G (p.Asp60Glu)	3	(Exon 3; +17)	Same as in WT
		c.319C>T (p.Arg107Trp)	3	(Exon 4; −35)	HEK293: shift in expression ratio: FL (minor) + Δ4q_53nts (minor) + Δ4 (major); HeLa: same as in WT
		c.325dup	3	(Exon 4; −29)	Same splice pattern as for c.319C>T
PMS2 exon 6	FL + three very minor alternative transcripts Δ6, Δ6p_49nts and Δ6p_52nts	c.538-3C>G^5^	2	na	Two transcripts: Δ6p_49nts (major) + Δ6p_52nts (minor)
		c.614A>C (p.Gln205Pro)	3	(+77)	HeLa: same as WT except no Δ6 (HEK293 nt)
		c.687T>C (p.=)	3	(−19)	HeLa: same as WT except no Δ6 (HEK293 nt)
PMS2 exon 8	FL including an artificial pseudoexon	c.823C>G (p.Gln275Glu)	3	(+20)	Two transcripts: Δ8p_20nts + Δ8p_8nts
		c.825A>G (p.=)	3	(+22)	Δ8p_22nts
		c.903G>T (p.Lys301Asn)	2	(−1)	Δ8
PMS2 exon 10	FL + very minor Δ10	c.989-2A>G^5^	1	na	Δ10
		c.989-1G>T^5^	1	na	Δ10
		c.1144+2T>A	1	na	Δ10
PMS2 exon 12	FL	c.2174+1G>A	1	na	HEK293: two transcripts, Δ12 + ▾12q_421nts; HeLa: one transcript, Δ12
PMS2 exon 14	FL + Δ14p_43 nts (subtle difference in expression between HEK293 and HeLa)	c.2445+1G>T	1	na	HEK293: two transcripts, ▾14q_85nts + Δ14p_43nts_▾14q_85nts; HeLa: three transcripts, Δ14 (major) + 2 transcripts as in HEK293 (both minor)
MSH2 exon 4	FL (Hela nt)	c.728G>A (p.Arg243Gln)	3	(−65)	Same as WT (Hela nt)
MSH2 exon 13	FL + minor Δ13 (Hela nt)	c.2006G>T (p.Gly669Val)	2	(+1)	Δ13 (Hela nt)
MSH6 exon 5	FL + minor Δ5 (Hela nt)	c.3438+1G>A	1	na	Δ5 (Hela nt)
MSH2 intron 1	No pseudoexon inclusion (Hela nt)	c.212-478T>G^5^	4	na	pseudoexon inclusion: intron 1 c.212-553_c.212-479 (Hela nt)
APC intron 4	No pseudoexon inclusion	c.532-941G>A^6^	4	na	Partial splice effect: normal transcript (=exon A+B only) + transcript with pseudoexon inclusion (▾167 nts from intron 4; as reported by Spier et al. 2012)
PKD2 intron 4	No pseudoexon inclusion (Hela nt)	c.1094+507A>G^5^	4	na	Same as in WT (Hela nt)
MSH2 intron 14	No pseudoexon inclusion	c.2459-834A>G	4	na	Same as in WT

WT, wildtype; FL, full-length (“normal”) mRNA transcript; nt, not tested; nts, nucleotides; na, not applicable; Δ, skip of complete or part of the exon (Δx, skip of exon x); ▾, inclusion of intronic sequence; p, acceptor-site shift, q, donor-site shift, p and q followed by the number of nts that are skipped or included.

1 Specification of minigene vectors and transfection cell lines used in splicing assays for each variant are provided in Table S3.

2 Detailed information on minigene amplicon design is provided in Table S1.

3 Nomenclature according to HGVS guidelines. Predicted protein changes for exonic variants are provided in brackets. The following reference sequences were used: NM_000249.3 for *MLH1*, NM_000535.5 for *PMS2*, NM_000251.2 for *MSH2*, NM_000179.2 for *MSH6*, NM_000038.5 for *APC*, and NM_000297.2 for *PKD2*.

4 Variants were classified in four categories according to their position relative to the consensus splice site regions. Category 1 = at the canonical −1, −2, +1, +2 position, category 2 = in the consensus splice site regions outside canonical positions, category 3 = exonic variants outside the consensus splice site regions, category 4 = deep intronic variants.

5 Variants from literature, created by SDM (*PMS2* c.538-3C>G en c.989-2A>G, Borras et al. 2013; *PMS2* c.989-1G>T, Sjursen et al. 2009; *MSH2* c.212-478T>G, Clendenning et al. 2011; *PKD2* c.1094+507A>G, Rossetti et al. 2012).

6 This variant, reported by Spier et al. 2012, was initially created by SDM. Because the patients carry the mutation in cis with a nearby SNP (*APC* c.532-845A>G; rs77939389), minigene assays were repeated with DNA from one of the patients, including the SNP in the minigene amplicon. Both assays showed the same results.

### Nomenclature of variants and description of splicing events

Variants were described according to the Human Genetic Variation Society (HGVS) approved guidelines (with the A of the initiation ATG codon as c.1; www.hgvs.org/mutnomen). The following Genbank reference sequences were used: NM_000249.3 for *MLH1*, NM_000535.5 for *PMS2*, NM_000251.2 for *MSH2*, NM_000179.2 for *MSH6*, NM_000038.5 for *APC*, and NM_000297.2 for *PKD2*. All new MMR variants in this study were submitted to the Leiden Open Variant Database (LOVD) for colorectal cancer variants (http://chromium.liacs.nl/LOVD2/colon_cancer/home.php).

Splicing events were described following conventions in literature (Thompson et al. 2015), using the following symbols: Δ (skipping of exonic sequence), ▾ (inclusion of intronic sequence), p (acceptor-site shift), q (donor-site shift). We did not quantify the relative expression of different transcripts produced in the minigene assay by the same variant or wild-type allele. Transcripts observed as a strong band on agarose gels are described as “major”; relatively weak bands on agarose gels or in sequence chromatograms, are described as “minor” transcripts.

### Splicing reporter minigene assays

All tests in this study were carried out using the previously described splicing reporter minigene vector pCAS2 (Gaildrat et al. 2010). Four variants were additionally tested using the minigene vector pSPL3b (Burn et al. 1995; Schneider et al. 2007). Amplicons were designed that included one or two exons, or the hypothesized pseudoexon in the case of a deep intronic variant, with a variable length of flanking 5′and 3′ intronic sequences (amplicon details and primer sequences are provided in Table S1). Genomic DNA from heterozygous patients or from control individuals was amplified using a high-fidelity DNA polymerase (either Phusion HF DNA polymerase, Thermo Scientific, Landsmeer, The Netherlands or Q5 HF DNA polymerase, New England Biolabs, Hitchin, UK). PCR products were cloned into the vector via the restriction sites BamHI and MluI for pCAS2, and XhoI and EcoRV for pSPL3b. Variants selected from literature were introduced into wild-type constructs using the Q5 SDM kit (New England Biolabs), following the manufacturer’s protocol (primer sequences for SDM in Table S2). Plasmid DNA was isolated using the GeneJET plasmid miniprep kit (Thermo Scientific), and the sequence of the construct was verified with vector-specific sequence primers surrounding the inserted amplicon (Table S1). The minor alleles of SNPs in cis with the investigated variant or present in the wild-type construct are recorded in Table S3A (variant constructs) and B (wild-type constructs). Wild-type and mutant constructs were transfected into HEK293 or HeLa cells using the FuGENE HD transfection reagent (Promega Benelux B.V., Leiden, The Netherlands) and 1 *μ*g of plasmid DNA. Cells, grown in 12-well plates to 50–90% confluence, were harvested 24–30 h after transfection, and total RNA was isolated using Nucleospin RNAII (Machery-Nagel, Düren, Germany). One microgram of RNA was used to synthesize cDNA (Omniscript™ RT Kit; Qiagen, Manchester, UK), using oligo-dT(15) primers (Promega). RT-PCR was performed using FastStart Taq DNA polymerase (Roche Diagnostics, Almere, The Netherlands) and vector-specific primers located in the flanking exons A and B (Table S1). Splice patterns of the minigene constructs were analyzed by electrophoresis on a 2% agarose gel, and by direct sequencing of the RT-PCR products. Specification of minigene vectors and transfection cell lines used for each variant is provided in Table S3A. Transfections were repeated on different time points for 12 of the 36 variant constructs (Table S3A); no major difference in splice patterns was observed between any of these independent transfections.

### Transcript analysis of patient RNA

For 30 of the 35 variants tested with minigene assays, data on patient RNA analysis were available (Table S4). These data were either obtained in our laboratory, reported in literature, or reported as a microattribution in the LOVD for colorectal cancer variants (v.2; build 35; http://chromium.liacs.nl/LOVD2/colon_cancer/; consulted on 10 April 2014). For the 18 variants tested in our laboratory, patient RNA analysis was performed using RNA isolated from short-term cultured peripheral blood lymphocytes (STCLs), with and without inhibition of NMD. NMD was inhibited by adding 100 mg/mL cycloheximide (Sigma-Aldrich, St. Louis, MO) 4–6 h before cell harvest (van der Klift et al. 2010; Vreeswijk and van der Klift 2012). RNA quality was assessed using the Agilent 2100 Bioanalyzer (Agilent Technologies, Middelburg, The Netherlands), and only samples with a RIN value between 7 and 10 were included in this study. RT-PCR was performed essentially as described in van der Klift et al. (2010), using either the Expand™ Long Template PCR system in combination with buffer 3 (Roche Diagnostics) or FastStart Taq DNA Polymerase (Roche Diagnostics). Primer sequences and additional information on RT-PCR conditions are provided in Table S5. For each RT-PCR amplicon, RNA from at least 10 control individuals was analyzed to check for alternative transcripts, with a minimum of two control samples included in an experiment together with a patient sample.

### Analytical classification of variants

The results from both minigene assays and patient RNA analyses were used to classify the 33 MMR variants following analytical classification guidelines developed previously for the interpretation of splicing alterations (Spurdle et al. 2008; Walker et al. 2013), except that we gave results obtained with minigene assays a more explicit role. In brief, a variant is considered pathogenic (class 5) when it shows aberrant splice products in patient RNA analysis resulting in a premature termination codon (PTC) or an in-frame deletion that disrupts known functional domains. Absence of full-length (FL) transcripts from the variant allele should be proven in patient RNA analysis or with a splicing reporter minigene assay. A variant is considered likely pathogenic (class 4), when the variant shows complete aberrant splicing in a minigene assay, but no patient RNA is available for confirmation. All other aberrant splice patterns, such as partial or incomplete aberrant splicing (i.e., still expressing some FL transcripts from the variant allele), and splice patterns showing merely a change in the ratio of FL and alternative transcript expression, were classified as VUS (class 3). Variants showing no aberrant splicing are designated as class 3 (missense variants) or class 2 (silent variants, intronic variants). Deep intronic variants showing no aberrant splicing in a minigene assay, but without patient RNA available for confirmation, are considered as VUS (and not class 2) because the degree of concordance between minigene assays and patient RNA analyses has not been fully established for this particular category of variants.

## Results

### Assessment of aberrant splicing for 35 variants using splicing reporter minigene assays

We tested 33 MMR gene variants, one *APC* variant and one *PKD2* variant for their effects on pre-mRNA splicing in a pCAS2 splicing reporter minigene assay, using 20 different minigene amplicon designs (Table1). All 20 wild-type constructs produced FL transcripts containing the constitutional exon(s) present in the amplicon design, but for 11 we also observed minor expression of alternative transcripts. Twenty of the 35 variant constructs tested showed complete aberrant splicing and five variants showed a partial splice effect, implying that these variant alleles still produced some normal FL transcripts (*APC* c.532-941G>A; *MLH1* c.793C>T; *MLH1* c.2103G>A; *PMS2* c.319C>T; and *PMS2* c.325dup). Ten of the 35 variant alleles did not produce any aberrant transcripts (Table1).

**Figure 1:**
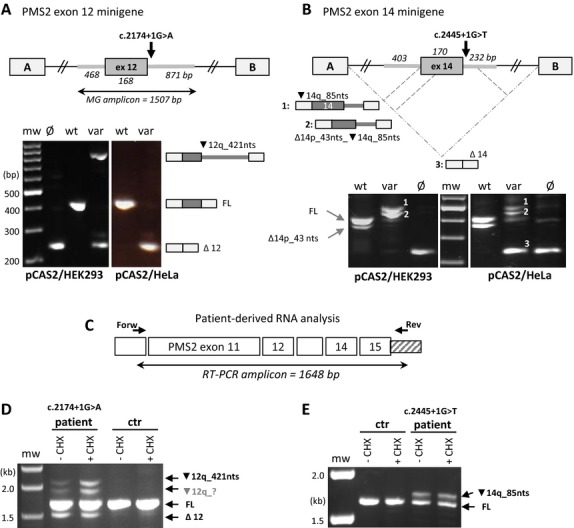
Differences in aberrant splice patterns produced by splicing reporter minigene (MG) assays and patient RNA analyses for *PMS2* c.2174+1G>A and c.2445+1G>T (NM_000535.5). (A–B) pCAS2 MG assays performed in HEK293 and HeLa cells for *PMS2* c.2174+1G>A (A) and c.2445+1G>T (B). Schematic minigene designs are depicted above, with pCAS2 exons A and B in white and the inserted *PMS2* amplicons in gray (box = exon, thick gray lines = *PMS2* intron sequences). RT-PCR fragments produced in the MG assay and separated by agarose gel electrophoresis are shown below. Schematic representation of transcripts corresponding with agarose bands are shown at the right (in panel A) or above (in panel B) the gel pictures. In panel B, transcripts 1, 2, and 3 correspond with fragments 1, 2, and 3 on agarose gel. (C) Schematic representation of RT-PCR design for patient-derived RNA analysis of both *PMS2* variants. (D–E) Patient RNA analysis for *PMS2* c.2174+1G>A (D) and *PMS2* c.2445+1G>T (E). The FL fragment (1648bp) contains *PMS2* exon 10–15 as depicted in panel C. We were not able to decipher the sequence of fragment ▾12q_?. MG, minigene; mw, molecular weight marker; wt, wild-type construct; var, variant construct; Ø, empty plasmid; FL, full-length transcript; ctr, control individual; +CHX and −CHX, with and without NMD inhibition by cycloheximide; nts, nucleotides; Δ, skip of complete or part of the exon (Δx, skip of exon x); ▾, inclusion of intronic sequence in transcript; p, acceptor-site shift, q, donor-site shift, p and q are followed by the number of nts that are skipped or included.

We tested 23 variants (12 different amplicons) in both HEK293 and HeLa cells. For all minigene assays the splice pattern produced in HEK293 was the same as in HeLa cells, except for two variant and one wild-type construct (confirmed in a second, independent transfection experiment). The variant allele *PMS2* c.2174+1G>A (Fig.1A) resulted in exon skip (Δ12) in both cell lines, but produced an additional aberrant transcript in HEK293 (▾12q_421nts). The variant allele *PMS2* c.2445+1G>T (Fig.1B) produced, in addition to two aberrant transcripts in both cell lines (▾14q_85nts and Δ14p_43nts_▾14q_85nts), a third aberrant transcript in HeLa cells (Δ14). The *PMS2* exon 3+4 wild-type construct produced splice patterns in which the ratio between expression of constitutive and alternative transcripts showed a major shift toward exon exclusion in HeLa compared to HEK293 cells (Fig. S1) suggesting that the assessment of splice effects for variants in this amplicon is less reliable in HeLa.

Four variants were also tested in the minigene vector pSPL3b (*MLH1* c.793C>A and c.793C>T; *PMS2* c.823C>G and c.825A>G). No differences in splice pattern were observed between these vectors for the PMS2 exon 8 variants (except for the artificial pseudoexon, see below). The *MLH1* exon 10 wild-type and the *MLH1* c.793C>T variant allele showed a minor shift towards exon exclusion in the pSPL3b minigene compared to pCAS2 (Fig. S2).

The *PMS2* exon 8 wild-type and variant pCAS2 constructs consistently produced transcripts that included an artificial pseudoexon in both HEK293 and HeLa transfections (Fig. S3). This 118bp pseudoexon consisted of the last 48 nts of the inserted *PMS2* intron 8 sequence, and the first 70 nts of the pCAS2 sequence downstream from the MluI cloning site. When tested in the pSPL3b vector (only transfected in HEK293; data not shown), we found an artificial 133bp pseudoexon inclusion for which the same 3′cryptic splice site in *PMS2* intron 8 (at position c.903+144) was used as in the pCAS2 assays. The artificial pseudoexon inclusion did not affect assessment of aberrant splicing for the three tested *PMS2* exon 8 variants.

### Comparison of splicing reporter minigene assays and patient RNA analyses

Results from patient RNA analyses were available for 30 variants (Table S4). We found 100% concordance between the minigene assay and patient RNA analysis in terms of whether or not a variant caused aberrant splicing (Table2). Twenty-three variants showed a complete or partial splice effect, whereas seven variants did not affect constitutional splicing.

**Table 2:** Splicing analysis and classification of 17 variants in the consensus splice site region (A), 14 exonic variants outside this region (B), and four deep intronic variants (C).

Variant^1^	Location^2^	Percent decrease of canonical ss strength (SSF, MES, NNS, HSF)^3^	Aberrant splicing in MG/patient RNA (different splice patterns)^4^	Effect of aberrant transcripts (MG and patient RNA)	FL transcript from variant allele (in MG)	Clinical classification in LOVD (v1.9:05/09/2013)^5^	Analytical classification based on RNA analyses^6^
*A. Variants in the consensus splice site regions*							
*Variants at the canonical −1, −2, +1, +2 positions (category 1)*							
MLH1 c.791-1G>C	Intron 9	100, 100, 100, 100	Yes/yes (no)	OOF exon skip (+ up regulation alternative transcripts in patient RNA)	No	5 [MLA; patient RNA analysis]	5
MLH1 c.1667+1del	Intron 14	25, 100, 90, 18	Yes/yes (no)	IF inclusion of 87 nts; no PTC	No	Not in LOVD	3 or 4 [disruption of functional domain by insertion of 29 amino acids should be proven experimentally]
PMS2 c.163+2T>C	Intron 2	4, 100, 100, 100	Yes/yes (no)	OOF exon skip	No	4 [absence FL not proven]	5
PMS2 c.989-2A>G (Borras et al. 2013)	Intron 9	100, 100, 100, 100	Yes/yes (no)	IF exon skip (in functional domain)	No	4 [absence FL not proven]	5
PMS2 c.989-1G>T (Sjursen et al. 2009)	Intron 9	100, 100, 100, 100	Yes/yes (YES)	IF exon skip (+ IF skip of 27 nts, patient RNA) (in functional domain)	No	Not in LOVD	5
PMS2 c.1144+2T>A	Intron 10	100, 100, 100, 100	Yes/yes (no)	IF exon skip (in functional domain)	No	4 [absence FL not proven]	5
PMS2 c.2174+1G>A	Intron 12	100, 100, 100, 100	Yes/yes (YES)	IF exon skip (in functional domain) + OOF inclusion of 421 nts (both IF and OOF transcripts cause partial NMD in patient RNA; in patient RNA a third, uncharacterized aberrant transcript present)	No	5 [patient RNA analysis]	5
PMS2 c.2445+1G>T	Intron 14	100, 100, 100, 100	Yes/yes (YES)	OOF inclusion of 85 nts (+ 2 OOF other transcripts in MG)	No	Not in LOVD	5
MSH6 c.3438+1G>A	Intron 5	100, 100, 100, 100	Yes/–	OOF exon skip	No	4 [no RNA analysis]	4 [MG shows absence of FL, but patient RNA analysis not performed]
*Variants in the consensus ss regions outside canonical positions (category 2)*							
MLH1 c.543C>G (p.=)	Exon 6 (−3)	4, 24, 71, 2	Yes/yes (no)	OOF exon skip	No	Not in LOVD	5
MLH1 c.545G>A (p.Arg182Lys)	Exon 6 (−1)	17, 98, 100, 14	Yes/yes (no)	OOF exon skip + OOF skip of 4 last exon nts	No	5 [patient RNA analysis]	5
MLH1 c.882C>T (p.=)	Exon 10 (−3)	4, 19, 11, 2	Yes/yes (no)	OOF exon skip (+ up regulation alternative transcripts in patient RNA)	No	5 [patient RNA analysis + MG]	5
MLH1 c.883A>G (p.Ser295Gly)	Exon 10 (−2)	11, 27, 67, 6	Yes/yes (no)	OOF exon skip (+ up regulation alternative transcripts in patient RNA)	No	5 [patient RNA analysis]	5
MLH1 c.2103G>A (p.=)	Exon 18 (−1)	17, 98, 89, 14	Yes/yes (no)	IF exon skip + minor IF skip of exon 17+18 (in functional domain)	Yes (minor)	4 [no RNA analysis]	3 ? [minor expression of FL transcript]
PMS2 c.538-3C>G (Borras et al. 2013)	Intron 5	12, 100, 100, 11	Yes/yes (YES)	OOF skip of first 49 exon nts (+ IF skip of complete exon 6 only in patient RNA) (+ OOF skip of first 52 nts only in MG)	No	Not in LOVD	5
PMS2 c.903G>T (p.Lys301Asn)	Exon 8 (−1)	16, 100, 100, 14	Yes/yes (no)	OOF exon skip	No	4 [absence FL not proven]	5
MSH2 c.2006G>T (p.Gly669Val)	Exon 13 (+1)	7, 23, 30, 5	Yes/yes (no)	OOF exon skip	No	5 [patient RNA analysis]	5

Variant^1^	Location^2^	Predicted effect on (cryptic) splice sites and/or ESE motifs^3^	Aberrant splicing in MG/patient RNA (different splice patterns)^4^	Effect of aberrant transcripts (MG and patient RNA)	FL transcript from variant allele (in MG)	Clinical classification in LOVD (v1.9:05/09/2013)^5^	Analytical classification based on RNA analyses^6^
*B. Exonic variants outside the consensus splice site regions (category 3)*							
MLH1 c.122A>G (p.Asp41Gly)	Exon 2 (+6)	Slight increase of weak 3′ css; no ESE change	No/no	Normal splicing	NA	5 [patient RNA analysis]	3 [our RNA analysis does not support pathogenicity; conflicting with Sharp et al. 2004]
MLH1 c.277A>G (p.Ser93Gly)	Exon 3 (−30)	Slight increase of weak 5′ css; decrease 1 ESE motif	No/–	Normal splicing	NA	3 [insufficient evidence]	3
MLH1 c.793C>A (p.Arg265Ser)	Exon 10 (+3)	Decrease of 1 ESE	Yes/yes (no)	OOF exon skip (+ up regulation alternative transcripts in patient RNA)	No	4 [MLA; no RNA analysis]	5
MLH1 c.793C>T (p.Arg265Cys)	Exon 10 (+3)	Increase of 1 ESE	Yes/yes (YES)	OOF exon skip (complete exon skip + up regulation of alternative transcripts in patient RNA; minor expression of FL in MG)	Yes (minor)	5 [patient RNA analysis]	3 ? [partial expression of FL in MG]
MLH1 c.1633A>G (p.Thr545Ala)	Exon 14 (-35)	Loss of 2 ESEs	No/no	Normal splicing	NA	3 [insufficient evidence]	3
PMS2 c.139C>T (p.=)	Exon 2 (-25)	Loss of 1 ESE	No/no	Normal splicing	NA	Not in LOVD	2
PMS2 c.180C>G (p.Asp60Glu)	Exon 3 (+17)	Loss of 1 ESE	No/–	Normal splicing	NA	2 [MLA]	3
PMS2 c.319C>T (p.Arg107Trp)	Exon 4 (−35)	Loss of 1 ESE; increase of 1 ESE	Yes/yes (no)	OOF exon skip + OOF skip of first 53 nts	Yes (minor)	Not in LOVD	3 [double deleterious mechanism?]
PMS2 c.325dup	Exon 4 (-29)	No change	Yes/yes (no)	OOF exon skip + OOF skip of first 53 nts	Yes (minor)	Not in LOVD	5 [double deleterious mechanism: splice effect + frame-shifting dup]
PMS2 c.614A>C (p.Gln205Pro)	Exon 6 (+77)	1 new ESE	No/no	Normal splicing	NA	3 [insufficient evidence]	3
PMS2 c.687T>C (p.=)	Exon 6 (−19)	2 new ESEs	No/no	Normal splicing		Not in LOVD	2
PMS2 c.823C>G (p.Gln275Glu)	Exon 8 (+20)	New 3′ss stronger than can.ss (in 3/4); loss of 1 ESE	Yes/–	OOF skip of first 20 nts + OOF skip of first 8 nts	No	Not in LOVD	4 [MG shows absence of FL, but patient RNA analysis not performed]
PMS2 c.825A>G (p.=)	Exon 8 (+22)	New 3′ss stronger than can.ss (4/4);	Yes/yes (no)	OOF skip of first 22 nts	No	Not in LOVD	5 [NB: in trans with PMS2 c.325dup in CMMR-D patient, Johannesma et al. 2011]
MSH2 c.728G>A (p.Arg243Gln)	Exon 4 (-65)	Loss of 6 ESEs	No/no	Normal splicing	NA	3 [insufficient evidence]	3

Variant^1^	Location	Splice site prediction at variant position (SSF, MES, NNS, HSF)^3^	Aberrant splicing in MG/patient RNA (different splice patterns)^4^	Effect of aberrant transcripts (MG and patient RNA)	FL transcript from variant allele (in MG)	Clinical classification in LOVD(v1.9:05/09/2013)^5^	Analytical classification based on RNA analyses^6^
*C. Deep intronic variants (category 4)*							
APC c.532-941G>A (Spier et al. 2012)	Intron 4	Increase of existing 5′css strength 2 nts downstream from variant [81>90; 6.6>8.7; 1.0=; 90.7>95.6]	Yes/yes (?)	OOF 167 nts pseudoexon inclusion	Yes	NA	3? [partial expression of FL in MG]
MSH2 c.212-478T>G (Clendenning et al. 2011)	Intron 1	Creation of new 5′ss at variant position [92.2; 7.3; 0.8; 87.0]	Yes/yes (no)	IF 75 nts pseudoexon inclusion,with PTC	No	5 [patient RNA analysis]	5
MSH2 c.2459-834A>G	Intron 14	Creation of new 5′ss at variant position [71.3; 1.8; 0.1; 75.1]	No/–	Normal splicing	NA	Not in LOVD	3 [no mRNA aberration in MG; needs confirmation in patient RNA]
PKD2 c.1094+507A>G (Rossetti et al. 2012)	Intron 4	Increase of existing 5′css strength 5 nts upstream of variant: [59.4>71.5; nr>7.,4; nr>0.9; 66.9>79.0];	No/no	Normal splicing	NA	NA	2 [no mRNA aberration in MG and patient RNA]

MG, minigene assay; ss, splice site; css, cryptic ss; 3′ss, acceptor ss; 5′ss, donor ss; OOF, out-of-frame; IF, in-frame; NA, not applicable; nr, not recognized; nts, nucleotides; FL, full-length (normal) transcript; MLA, multifactorial likelihood analysis; LOVD, (http://chromium.liacs.nl/LOVD2/colon_cancer/home.php); CMMR-D, constitutional mismatch repair-deficiency; NMD, nonsense-mediated mRNA decay.

1 Nomenclature according to HGVS guidelines. The following reference sequences were used: NM_000249.3 for *MLH1*, NM_000535.5 for *PMS2*, NM_000251.2 for *MSH2*, NM_000179.2 for *MSH6*, NM_000038.5 for *APC*, and NM_000297.2 for *PKD2*. Nomenclature at RNA level (as found in patient RNA analysis) according to HGVS guidelines is provided in Table S4.

2 The position of exonic variants is given relative to the acceptor (+) or donor (−) splice site.

3 In silico splice site prediction was performed using software programs available through the integrated software package Alamut version 2.0 (Interactive Biosoftware, Rouen, France), with thresholds set at zero: Splice Site Finder (SSF), Splice Site prediction by Neural Network (NNS), MaxEntScan (MES), Human Splicing Finder (HSF). Furthermore, ESEfinder 3.0, with default settings, was used to predict loss or gain of exonic splice enhancer (ESE) motifs (also applied through Alamut version 2.0). Decrease in splice site (ss) strength (Table2A) was calculated as follows: [(ss score_WT – ss score_MUT)/ss score WT]^*^100%.

4 Splicing analysis data for MG assays as presented in Table1, for patient RNA as reported in Table S4. In brackets whether a major difference in splice pattern is observed between both tests.

5 The LOVD uses a 5-tier clinical variant classification, applying criteria as described in Thompson et al. 2014. Class 1 = not pathogenic/low clinical significance; class 2 = likely not pathogenic/little clinical significance; class 3 = uncertain; class 4 = likely pathogenic; class 5 = pathogenic. We consulted the LOVD on April 10, 2014. Justification, given at LOVD, is written in brackets.

6 A combination of patient RNA analysis and minigene assay data was used for analytical classification. Please note that only clinical variant classification, combining all existing evidence, is linked to specific recommendations concerning surveillance and clinical management of a family (Sijmons et al. 2013). Class 5 = PTC-introducing transcript, or in-frame deletion in functionally important domain; absence of FL from variant allele proven. A variant with supporting minigene assay data only will not be classified higher than likely pathogenic (class 4). Additional justification is written in brackets.

**Figure 2:**
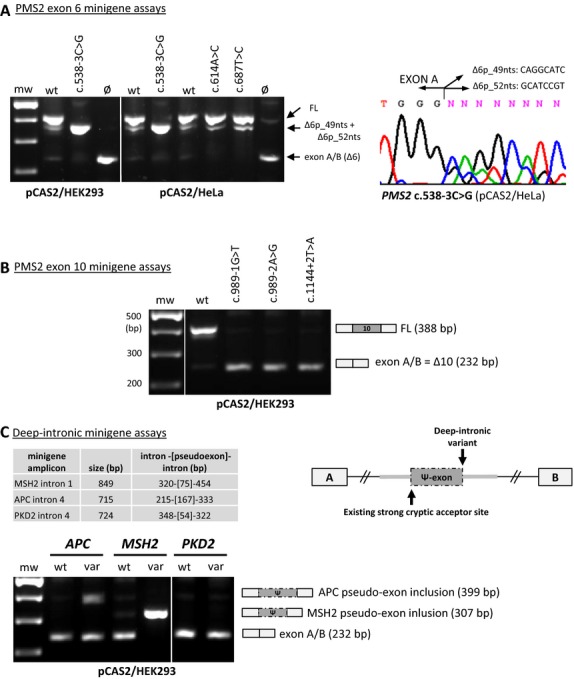
Splicing reporter pCAS2 minigene (MG) assays for variants in the *PMS2* exon 6 and exon 10 minigene amplicons (NM_000535.5), and for deep-intronic variants reported in literature. (A) The *PMS2* exon 6 minigene wild-type allele produces major FL transcripts and three very minor alternative transcripts Δ6p_49nts, Δ6p_52nts, and Δ6, hardly visible on gel. (NB: in lymphocyte RNA we only observed the alternative transcript Δ6p_49nts). The *PMS2* c.538-3C>G minigene, tested in both HEK293 and HeLa cells, shows complete aberrant splicing, consisting of Δ6p_49nts and Δ6p_52nts (sequence chromatogram at right), a splice pattern different from the one reported by Borras et al. 2013, in patient RNA. *PMS2* c.614A>C (p.Gln205Pro), and *PMS2* c.687T>C (p.=), both tested only in HeLa, show a subtle difference in alternative expression (lacking Δ6) in comparison to the wild-type minigene allele (sequence chromatograms available on request). (B) *PMS2* c.989-1G>T, c.989-2A>G, c.1144+2T>A, tested with the pCAS2 *PMS2* exon 10 minigene transfected in both HEK293 and HeLa, all show complete skip of exon 10 (HeLa transfections not shown). For *PMS2* c.989-1G>T, Sjursen et al. 2009, observed an additional aberrant transcript with skip of the first 27 nts of exon 10 in RNA isolated from a homozygous patient. (C) pCAS2 minigene assays performed for deep-intronic variants reported in literature (NM_000038.5:*APC* c.532-941G>A in Spier et al. 2012; NM_000251.2:*MSH2* c.212-478T>G in Clendenning et al. 2011; NM_000297.2:*PKD2* c.1094+507A>G in Rossetti et al. 2012). The APC variant was tested in both HEK293 (see agarose gel) and HeLa (same splice pattern, not shown); MSH2 and PKD2 variants were only tested in HEK293. At the right, a representative minigene design for a hypothesized pseudoexon is depicted. MG assays for *MSH2* c.212-478T>G, showing complete pseudoexon inclusion, and for *PKD2* c.1094+507A>G, showing no aberrant splicing, are concordant with reported patient RNA analyses (Clendenning et al. 2011; Rossetti et al. 2012). The *APC* c.532-941G>A minigene produces normal transcripts in addition to aberrant transcripts with the same pseudoexon included as reported for patient RNA (Spier et al. 2012). Partial splicing has not been investigated for this variant in RNA isolated from the patient. MG, minigene; mw, molecular weight marker; wt, wild-type construct; var, variant construct; Ø, empty plasmid; FL, full-length transcript; bp, base pair; nts, nucleotides; Δ, skip of complete or part of the exon; ▾, inclusion of intronic sequence in transcript; p, acceptor-site shift, p is followed by the number of nts that are skipped.

However, for four variants showing aberrant splicing, major differences in aberrant splice pattern were observed between the minigene and patient RNA assays (*PMS2* c.538-3C>G, *PMS2* c.989-1G>T, *PMS2* c.2174+1G>A, *PMS2* c.2445+1G>T; Figs.1, 2).

*PMS2* c.538-3C>G showed two aberrant transcripts, an in-frame skip of exon 6 and a deletion of the first 49bp of exon 6, in patient RNA isolated from lymphocytes cultured with NMD inhibitors (Borras et al. 2013). We then tested this variant in the minigene assay and found two aberrant transcripts, with deletion of the first 49bp and the first 52bp of exon 6, respectively, but no transcript with a skip of the entire exon 6 (Fig.2A). Sjursen et al. (2009) detected two aberrant transcripts (*PMS2* Δ10 and Δ10p_27nts) in mRNA from a patient homozygous for *PMS2* c.989-1G>T. In our minigene assay this variant produced only the whole exon 10 skip transcript, the same result found for *PMS2* c.989-2A>G and c.1144+2T>A (Fig.2B).

Using our RNA-based mutation scanning protocol for *PMS2* (van der Klift et al. 2010), we found aberrant splice products in cultured lymphocyte RNA for the heterozygous *PMS2* variants c.2174+1G>A and c.2445+1G>T (Fig.1C). For *PMS2* c.2174+1G>A, we observed three aberrant splice products in patient RNA, whereas the minigene variant allele revealed only two of these aberrant transcripts in HEK293 (Δ12; ▾12q_421nts) and only one (Δ12) in HeLa cells (Fig.1A). The other variant, *PMS2* c.2445+1G>T, showed one aberrant transcript, with retention of 85bp of flanking intronic sequence (▾14q_85nts) in patient RNA. In the minigene assays, one (in HEK293) or two (in HeLa) extra aberrant transcripts were observed (Δ14p_43nts_▾14q_85nts in both Hek293 and HeLa; Δ14 in HeLa only; Fig.1B). Remarkably, both the variant as well as the wild-type minigene allele produced an alternative transcript using a weak cryptic acceptor splice site in exon 14 (*PMS2* c.2318) located 43 nucleotides downstream from the canonical splice site. We never observed the use of this cryptic splice site in lymphocyte RNA from patients or controls.

For one variant, MLH1 c.793C>T, we found a minor discrepancy between patient RNA analysis and our minigene assays. Microattributions to the LOVD (http://chromium.liacs.nl/LOVD2/colon_cancer/home.php) by Holinsky-Feder & Laner (submitter ID LOVD00057; microAttrID 200066) and by Leung (submitter ID 0000-0001-8614-4619; microAttrID 200068) reported complete aberrant splicing observed in patient RNA analysis, whereas we observed a partial splice effect in the minigene assays. The variant allele, tested in both pCAS2 and pSPL3b minigenes and in both HEK293 and HeLa transfections, shows minor expression of the FL transcript in addition to the major aberrant transcript with exon 10 exclusion (Fig. S2). Tournier et al. (2008) also reported partial aberrant splicing for this variant and quantified the exon 10 exclusion as ∼60% in pCAS and nearly 100% in pSPL3, both transfected in HeLa.

### Analytical variant classification using minigene assay and patient RNA analysis data, compared to clinical variant classification reported by InSiGHT

Recently, the InSiGHT applied a 5-tier scheme to the clinical classification of all 2360 constitutional MMR gene variants present in the InSiGHT Colon Cancer Gene Variant Database at the end of 2012 (Thompson et al. 2014). Designated classes are published at the LOVD website (http://chromium.liacs.nl/LOVD2/colon_cancer/home.php) that hosts the InSiGHT database, and justification for each classification is reported (http://insight-group.org/variants/classifications/). Variants are classified as not or likely not pathogenic (class 1 and class 2, respectively), uncertain (class 3), or (likely) pathogenic (class 4 and class 5), using criteria developed by the InSiGHT Variant Interpretation Committee (Thompson et al. 2014). These “InSiGHT” criteria integrate several lines of evidence including guidelines specifically developed for the interpretation of splicing alterations (Spurdle et al. 2008; Walker et al. 2013). We compared these clinical classifications with our analytical classifications (see Material and Methods) for the 33 MMR gene variants in the study (Table2).

Twelve of the 33 MMR variants were not reported by InSiGHT at the moment of website consultation (April 10, 2014; Table2). Seven of these variants showed complete aberrant splicing and were classified as pathogenic based on a combination of minigene assay and patient RNA analysis data, or as likely pathogenic based on minigene data alone (*PMS2* c.823C>G; no patient RNA available). However, following current InSiGHT criteria the latter variant is clinically classified as a VUS (class 3). Two variants in *PMS2* exon 4 (*PMS2* c.319C>T; p.Arg107Trp, and *PMS2* c.325dup; Fig. S1) showed a similar shift in expression from FL to the two alternative transcripts (Δ4 and Δ4q_53nts) in both minigene and patient RNA analysis. The variant alleles still produced some FL transcripts (partial splice effect) and possibly act through a double deleterious mechanism: while part of the mRNA is deleterious due to a major shift to alternative transcripts that introduce a PTC, the residual FL transcripts are deleterious due to a frameshift (*PMS2* c.325dup; a priori class 5) or a possibly functional missense mutation (*PMS2* c.319C>T, p.Arg107Trp). Three variants (two silent and one deep intronic) did not show any aberrant splicing. We classified the silent variants as likely not pathogenic (class 2), and the deep intronic variant (*MSH2* c.2459-834A>G) as a VUS.

For 13 of the 33 variants our pathogenicity assessments were concordant with the InSiGHT classifications: for seven variants our splicing analyses confirmed previously reported data on splicing, whereas for five variants, the splicing analysis results confirm the LOVD classifications that were justified using other evidence such as multifactorial likelihood analysis (Table2). RNA analysis of PMS2 c.180C>G did not show aberrant splicing, classifying this variant as a VUS; multifactorial likelihood analysis (MLA), however, classified this variant further as likely not pathogenic.

**Figure 3:**
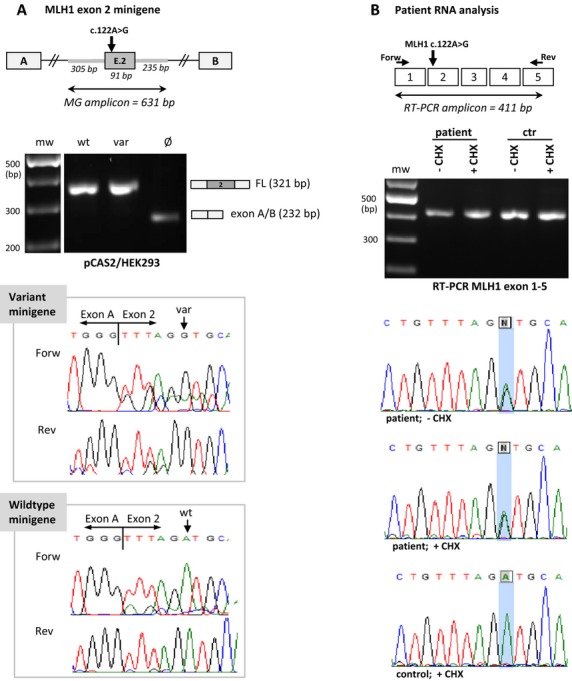
Splicing analysis for *MLH1* c.122A>G (NM_000249.3) with a pCAS2 minigene transfected in HEK293 cells (see agarose gel) and HeLa (not shown) and with RT-PCR of MLH1 exon 1-5 on patient RNA isolated from cultured lymphocytes with (+CHX) and without (-CHX) inhibition of NMD. No aberrant splicing is observed in neither of the tests which is in conflict with complete exon 2 skipping reported for this variant by Sharp et al. 2004. (A) Schematic design of the *MLH1* exon 2 minigene is shown at the top. Agarose gel electrophoresis and sequence chromatograms of RT-PCR fragments produced by the variant and the wild-type minigene assay are shown below. In the sequence chromatograms an alternative transcript Δ2p_5nts runs through the FL transcript (minor up regulation of Δ2p_5nts observed in the variant assay). (B) Schematic design, agarose gel electrophoresis, and sequence chromatograms from the patient RNA analysis of the heterozygous *MLH1* c.122A>G variant are shown from top to bottom, respectively. Reverse complement sequences performed with the Reverse primer in *MLH1* exon 5 are shown. MG, minigene; bp, base pair; mw, molecular weight marker; wt, wild-type construct; var, variant construct; Ø, empty plasmid; FL, full-length transcript; ctr, control individual; +CHX and −CHX, with and without NMD inhibition by cycloheximide; nts, nucleotides; Δ, skip of complete or part of the exon; p, acceptor-site shift, p is followed by the number of nts that are skipped; Forw, sequenced with Forward primer; Rev, sequenced with Reverse primer.

For eight of the 33 variants our splicing analyses data suggest a classification that differs from that of InSiGHT (classification version v1.9: 5/09/2013). Four ‘likely pathogenic’ variants can now be classified as ‘pathogenic’ because minigene assays showed absence of FL expression from the variant allele. One InSiGHT class 4 variant (*MLH1* c.793C>A) for which no RNA data were available can now be designated as class 5 based on our minigene and patient RNA analyses. Remarkably, the *MLH1* c.793C>A variant caused complete aberrant splicing, whereas the *MLH1* c.793C>T variant allele still produced some FL transcript in the minigene assay (Fig. S2), not supporting the class 5 status justified because absence of FL transcripts was reported in patient RNA (as mentioned above). For MLH1 c.2103G>A we found some residual FL transcript produced by the variant allele, both with the minigene assay as in patient RNA. Although expression of the FL transcript seems very minor, strictly following the guidelines this variant should be analytically classified as a VUS. Finally, for one variant, *MLH1* c.122A>G, we could not confirm the pathogenicity that was based on aberrant splicing reported by Sharp et al. (2004). Neither our minigene assay nor our patient RNA analysis showed skipping of *MLH1* exon 2 (Fig.3). Absence of aberrant splicing for this variant was confirmed by another clinical diagnostic laboratory using a fresh blood sample from the same patient (personal communication dr. M. Blok; Department of Clinical Genetics, Maastricht University Medical Center, The Netherlands).

## Discussion

Although patient RNA analysis is the preferred approach to the evaluation of potentially spliceogenic variants in genes that are expressed in peripheral blood lymphocytes, such as the MMR and BRCA genes, splicing reporter minigene assays represent an interesting alternative in some situations. The performance of minigene assays compared to patient RNA analyses has been investigated in several studies (Bonnet et al. 2008; Tournier et al. 2008; Thery et al. 2011; Steffensen et al. 2014), together for 142 unique MMR gene and BRCA1/2 gene variants (this study included; Table3). Overall, high concordances were found between the two methods, with only a few variants showing differences in aberrant splicing profiles. In our study four of the 22 spliceogenic MMR variants showed major differences in aberrant splice patterns, meaning that one or more extra aberrant transcripts were produced by the variant allele either in the minigene assay or in patient RNA (Figs.1, 2). The presence of additional aberrant transcripts has also been reported for four BRCA1 and two BRCA2 variants (Bonnet et al. 2008; Acedo et al. 2012; Steffensen et al. 2014; Table3). However, for all 10 variants the observed differences in aberrant transcript profile between both tests did not influence their classification as pathogenic or likely pathogenic. Minor differences in splice effect between minigene assays and patient RNA analyses were also reported, such as for the four variants that show weak exon exclusion in one of both tests (Table3; Tournier et al. 2008; Thery et al. 2011; Steffensen et al. 2014), or the partial versus complete exon skip shown for *MLH1* c.793C>T in minigene assay and patient RNA analysis, respectively (this study).

**Table 3:** Concordance between splice effects shown with minigene assay (MG) and patient RNA analysis for 142 unique MMR gene and BRCA gene variants^1^ reported in literature and in this study.

Publication	Gene(s)	Source of patient RNA	Minigene vector/transfected cell line	Number of variants^2^ [with splice effect]	Concordance between MG assay and patient RNA showing a splice effect or not	Variants showing major difference in aberrant splice pattern between MG assay and patient RNA
Auclair et al. (2006)	MLH1 and MSH2	LCLs with NMD inhibition	pTARGET/COS-7	5 [2]	5 of 5	None
Tournier et al. (2008)	MLH1 and MSH2	PAXgene blood + reports from literature	pCAS/HeLa (5 variants also in DLD1 and COS-7 cells; same 5 variants also in pSPL3/HeLa)	37 [12]	35 of 37 (two variants MSH2 c.2006-6T>C and MLH1 c.790+10A>G with minor splice effect in MG, low expression of transcripts with exon skip, not observed in patient RNA)	None
Bonnet et al. (2008)	BRCA1 and BRCA2	PAXgene blood	pCAS/HeLa	20 [7]	20 of 20	2 variants show one aberrant transcript in patient RNA + one extra in MG assay (BRCA1 c.4987-5T>A; BRCA2 c.7805G>C)
Sanz et al. (2010)	BRCA1 and BRCA2	PBLs	pSPL3/HeLa and MCF10A	4 [3]	4 of 4	None
Thery et al. (2011)	BRCA1 and BRCA2	PAXgene blood	pCAS/HeLa	30 [9]	29 of 30 (BRCA2 c.68-7T>A in MG assay no effect, in patient RNA enhanced exon 3 skip)	None
Acedo et al. (2012)	BRCA2	PBLs	pSPL3/HeLa	2 [1]	2 of 2	1 variant shows two aberrant transcripts in patient RNA + one extra in MG assay (BRCA2 c.8488-1G>A)
Steffensen et al. (2014)	BRCA1	PBLs and LCLs	pSPL3/COS-7	24 [17]	23 of 24 (BRCA1 c.5074+6C>G in MG assay enhanced exon 17 skip, not observed in patient RNA)	3 variants show one aberrant transcript in patient RNA + one extra in MG assay (BRCA1 c.441+1G>A; c.4986+6T>G; c.5074+1G>T)
Our study	MLH1, PMS2, MSH2 and MSH6	STCLs with NMD inhibition + reports from literature/LOVD	pCAS2/HEK2913 and/or HeLa	28 [22]	28 of 28	2 variants show extra aberrant transcripts in patient RNA (PMS2 c.989-1G>T and c.2174+1G>A); 1 shows an extra transcript in MG assay (PMS2 c.2445+1G>T); 1 shows an extra transcript in both patient RNA and in MG assay (PMS2 c.538-3C>G)

NMD, nonsense-mediated mRNA decay; LCLs, lymphoblastoid cell lines; PBLs, peripheral blood lymphocytes; STCLs, short-term cultured lymphocytes; LOVD, Leiden open source variant database (http://chromium.liacs.nl/LOVD2/colon_cancer/home.php).

1 Nomenclature according to HGVS guidelines. The following reference sequences were used for gene variants mentioned in this table: NM_000249.3 for *MLH1*, NM_000251.2 for *MSH2*, NM_000535.5 for *PMS2*, NM_007294.3 for *BRCA1*, NM_000059.3 for *BRCA2*.

2 Number of variants for which results from both minigene assays and from patient RNA analyses are presented in the referred publication (total 150, including 142 different variants; six variants were tested in two or three studies). Numbers of variants that show aberrant splicing are written in brackets (total 73, including 71 different variants).

Variations in splice pattern between different minigene vectors or transfection cell lines have also been noted (Tournier et al. 2008). However, reproducibility of the minigene assay between different vectors and cell lines has not been extensively investigated. We tested four variants present in two minigene amplicons in both pCAS2 and pSPL3b and observed only minor shifts in expression of FL and aberrant transcript for the wild-type *MLH1* exon 10 and one variant in this exon (*MLH1* c.793C>T; Fig. S2). Similar shifts in expression ratios were shown by Tournier et al. (2008) for four of five variant alleles (also including *MLH1* c.793C>T) and two of four wild-type alleles tested in both pCAS (version 1) and a modified pSPL3 vector. Comparing two different transfection cell lines, we did find some major differences in transcript profiles. Of the 23 tested variant alleles, two produced an extra aberrant transcript (*PMS2* c.2174+1G>A in HEK293, and *PMS2* c.2445+1G>T in Hela; Fig.1), and one of the 12 wild-type constructs showed a major shift in expression from FL to alternative exon skip in HeLa cells compared to HEK293 (*PMS2* exon 3+4 amplicon, Fig. S1). Such differences between cell lines, accept for minor quantitative differences, were not reported by Tournier et al. (2008) who tested five variants in three different cell lines (HeLa, DLD1, COS-7), or Sanz et al. (2010), who tested three splice variants in HeLa and in MCF10A cells.

All these discrepant splice patterns, major and minor, observed between minigene assays and patient RNA analyses, and between different vectors and transfection cell lines used in the minigene assays, may be due to cell-type-specific differences in expression of splice factors and splice regulatory factors, or to differences in genomic context caused by, for example, the limited minigene amplicon length allowing only one or two exons to be included in the minigene assay (Baralle et al. 2006). Also differences in experimental protocols, for minigene assays as well as for patient RNA analyses, can be responsible for these inconsistencies. However, the overall concordance between patient RNA analyses and minigene assays in the assessment of aberrant splicing is nearly 100%, as shown in previous studies and confirmed in our study (Table3). Both tests assessed 71 of the 142 unique variants as spliceogenic and 67 as nonspliceogenic. Four variants assessed as nonspliceogenic in one test, showed a minor splice effect in the other test.

Although high concordance for splicing analysis is demonstrated between splicing reporter minigene assays and patient RNA analyses, the current consensus is that minigene results should always be checked against patient RNA (Buratti et al. 2013). This appears justified in light of the differences in splice pattern between the minigene assay and patient-derived lymphocyte mRNA for a proportion of spliceogenic variants. However, it is worth remembering that results from patient RNA analyses may also be biased by protocol limitations, confounding alternative transcripts, and cell-type-specific differences in splice profiles between lymphocytes and the affected tissue (Whiley et al. 2014). Nevertheless, the recently published InSiGHT criteria for clinical variant classification (Thompson et al. 2014) currently accept pathogenic (class 5) status for a splice variant only when patient RNA is used to prove complete aberrant splicing. A variant with such splice effect found in a minigene assay, and for which additional clinical evidence is not yet available, is currently classified as VUS, except for variants at the canonical ±1, ±2 positions that are classified a priori as likely pathogenic (class 4). We would now argue, on the basis of almost 100% concordance between minigene assays and patient RNA analyses for at least 142 unique MMR and BRCA gene variants (Table3) in the assessment of whether a variant causes aberrant splicing or not, that minigene splicing assay data alone is sufficient to justify the clinical classification of splice variants as likely pathogenic (class 4). A prerequisite, however, is that explicit guidelines for assay design and data reporting should be developed. On the basis of our experience for instance, we recommend that amplicons be tested (both wild-type and variant alleles) in additional cell lines when the wild-type construct shows unexpected major exclusion of constitutional exons. Also, it should be kept in mind that minigene vectors such as used in our study are not suitable for the analysis of variants in the first and last exon and intron. Adapted minigenes should be designed for such variants (e.g., Naruse et al. 2009; Grodecka et al. 2014). Genomic regions where multiple exons are involved in alternative splicing, as is the case for MLH1 exon 9, 10, and 11, are also more challenging for a representative minigene design (Bianchi et al. 2011; Thompson et al. 2015).

For one of the variants in our study (*MLH1* c.122A>G) we could not confirm previously reported aberrant splicing. In contrast to Sharp et al. (2004) who observed complete skipping of exon 2 associated with *MLH1* c.122A>G, our minigene assay and patient RNA analysis did not show an effect of this variant on splicing, except for a minor upregulation of the alternative transcript Δ2p_5nts (Fig.3). A possible explanation for these conflicting results could be the presence of an undetected mutation responsible for the exon 2 skip in the RNA of the *MLH1* c.122A>G carrier studied by Sharp et al. Clinical classification of this missense variant (p.Asp41Gly), reported as pathogenic in LOVD based on aberrant splicing, however, should now be reconsidered.

All 17 variants in the consensus splice site regions, that would have been selected for experimental RNA analysis using in silico splice prediction tools, showed indeed aberrant splicing in the RNA analyses (Table2A). Although splice prediction is highly reliable for variants in these consensus regions, the exact molecular nature of the aberrant splicing can only be determined by experimental analysis. Even for variants at the canonical splice sites, classified a priori as likely pathogenic by the InSiGHT criteria, experimental RNA analysis is advised. For example, the MLH1 c.1667+1del in our study did not cause the expected out-of-frame skip of exon 14 but instead showed inclusion of 87 bp intronic sequence predicted to result in an in frame insertion of 29 amino acids in the protein. Further evidence is needed to establish pathogenicity for this variant. Our study included 14 exonic variants outside the consensus splice site regions and four deep intronic variants. Eight of these 14 exonic variants (Table2B) did not show a splice effect, despite the fact that loss of one to six ESE motifs was predicted by ESEfinder 3.0 for four of the variants, in keeping with the poor specificity previously associated with this prediction tool (Houdayer et al. 2012). Four exonic variants that did cause complete or partial aberrant splicing (*MLH1* c.793C>A and c.793C>T; *PMS2* c.319C>T and c.325dup) may have disrupted SREs. However, ESE loss was not convincingly predicted by ESEfinder 3.0. In contrast, two exonic variants predicted to create new splice sites stronger than the canonical acceptor site of *PMS2* exon 8 (*PMS2* c.823C>G and c.825A>G) indeed caused aberrant splicing through splice site shifts.

In an era of next-generation sequencing, deep intronic variants are a growing category of VUS and may cause aberrant splicing through pseudoexon inclusion (Dhir and Buratti 2010). Concordances between in silico prediction and experimental analysis and between patient RNA analyses and minigene assays have not yet been extensively investigated for this type of variant. Although in silico tools can predict splice-site creation or activation, actual usage resulting in pseudoexon inclusion is still hard to predict; not only is the proximity of other splicing and splice regulatory motifs important, other factors also play a role (e.g., the presence of inhibitory RNA secondary structures that influence in vivo splicing). For three of the deep intronic variants investigated, the minigene assay results were concordant with reported patient RNA analyses (Table2C; Fig.2). One of these variants (*APC* c. 532-941G>A) showed partial splicing in the minigene assays. Whether this partial splice effect is caused by the limited genomic context of the minigene or that it reflects the true nature of aberrant splicing in the patients RNA could not be established because normal mRNA expression from the variant allele was not investigated in patient RNA analysis (Spier et al. 2012).For one deep intronic variant (*MSH2* c.2459-834A>G) that showed no aberrant splicing, patient RNA was unavailable. Because concordance between minigene and patient RNA analysis results has not yet been established for this particular category of variants, this variant remains a VUS.

In our study, we confirmed high concordance between patient RNA analyses and minigene assays in the assessment of aberrant splicing. Given that a fraction of the variants for which we could compare minigene assays and patient RNA analyses show differences in the exact splice pattern between tests, we concur that variants should not be classified as definitely pathogenic based on minigene assays alone. However, in light of the high concordance in the assessment whether a variant causes aberrant splicing, now recorded for 142 unique MMR and BRCA gene variants (Table3), we suggest that minigene assays showing complete aberrant and frame-shifting splice effects warrant upgrading a VUS (class 3) to a likely pathogenic variant (class 4), even in the absence of patient RNA. This reclassification is not merely academic but would have important consequences for the patient, as carriers of a likely pathogenic variant and a pathogenic variant fall under the same surveillance recommendations (Sijmons et al. 2013). In conclusion, minigene splicing assays can make a valuable contribution to variant classification and should therefore be incorporated in clinical diagnostic practice.

## Supporting Information

**Figure S1.** Splicing reporter minigene assays (A) and patient RNA analyses (B) for *PMS2* c.319C>T and c.325dup. **Figure S2.** Minor quantitative differences in exon exclusion for the wild-type MLH1 exon 10 and the MLH1 c.793C>T constructs in the pCAS2 vector compared with pSPL3b. **Figure S3.** Artificial pseudoexon inclusion in transcripts produced by *PMS2* exon 8 wild-type and variant alleles in pCAS2 transfected in HEK293 (in HeLa transfections the same splice patterns were observed, data not shown). **Table S1.** Minigene amplicons: design and primer sequences. **Table S2.** Primers used for site-directed mutagenesis (SDM) in pCAS2 wild-type constructs. **Table S3.** (A) Specification of minigene vectors and transfection cell lines used in splicing assays for each variant tested in this study. (B) Minor alleles of SNPs present in the wild-type constructs used in this study. **Table S4.** Information on patient RNA analysis (RT-PCR) of the 35 variants described in this study. **Table S5.** Primer sequences used for RT-PCR in the patient RNA analyses performed in our laboratory (LUMC; research section).
