# Supplementary material for: Splicing analysis for exonic and intronic mismatch repair gene variants associated with Lynch syndrome confirms high concordance between minigene assays and patient RNA analyses
Source: Mol Genet Genomic Med. 2015 Apr 23;3(4):327–45. doi: 10.1002/mgg3.145 (PMC4521968; doi:10.1002/mgg3.145)
Supplement: Supplementary file 1 — Figure S1. Splicing reporter minigene assays (A) and patient RNA analyses (B) for PMS2 c.319C>T and c.325dup. Figure S2. Minor quantitative differences in exon exclusion for the wild-type MLH1 exon 10 and the MLH1 c.793C>T constructs in the pCAS2 vector compared with pSPL3b. Figure S3. Artificial pseudoexon inclusion in transcripts produced by PMS2 exon 8 wild-type and variant alleles in pCAS2 transfected in HEK293 (in HeLa transfections the same splice patterns were observed, data not shown). Table S1. Minigene amplicons: design and primer sequences. Table S2. Primers used for site-directed mutagenesis (SDM) in pCAS2 wild-type constructs. Table S3. (A) Specification of minigene vectors and transfection cell lines used in splicing assays for each variant tested in this study. (B) Minor alleles of SNPs present in the wild-type constructs used in this study. Table S4. Information on patient RNA analysis (RT-PCR) of the 35 variants described in this study. Table S5. Primer sequences used for RT-PCR in the patient RNA analyses performed in our laboratory (LUMC; research section). [file mgg30003-0327-sd1.docx]

**Supporting information**

**Table of contents:**

- **Supp. Figure S1:** Splicing reporter minigene assays (A) and patient RNA analyses (B) for *PMS2* c.319C>T and c.325dup.
- **Supp. Figure S2:** Minor quantitative differences in exon exclusion for the wildtype MLH1 exon 10 and the MLH1 c.793C>T constructs in the pCAS2 vector compared with pSPL3b.
- **Supp. Figure S3:**  Artificial pseudo-exon inclusion in transcripts produced by *PMS2* exon 8 wildtype and variant alleles in pCAS2 transfected in HEK293 (in HeLa transfections the same splice patterns were observed, data not shown).
- **Supp. Table S1:** Minigene amplicons: design and primer sequences
- **Supp. Table S2:** Primers used for site-directed mutagenesis (SDM) in pCAS2 wildtype constructs
- **Supp. Table S3a:** Specification of minigene vectors and transfection cell lines used in splicing assays for each variant tested in this study.
- **Supp. Table S3b:** Minor alleles of SNPs present in the wildtype constructs used in this study**.**
- **Supp. Table S4:** Information on patient RNA analysis (RT-PCR) of the 35 variants described in this study
- **Supp. Table S5:** Primer sequences used for RT-PCR in the patient RNA analyses performed in our lab (LUMC; research section).

**
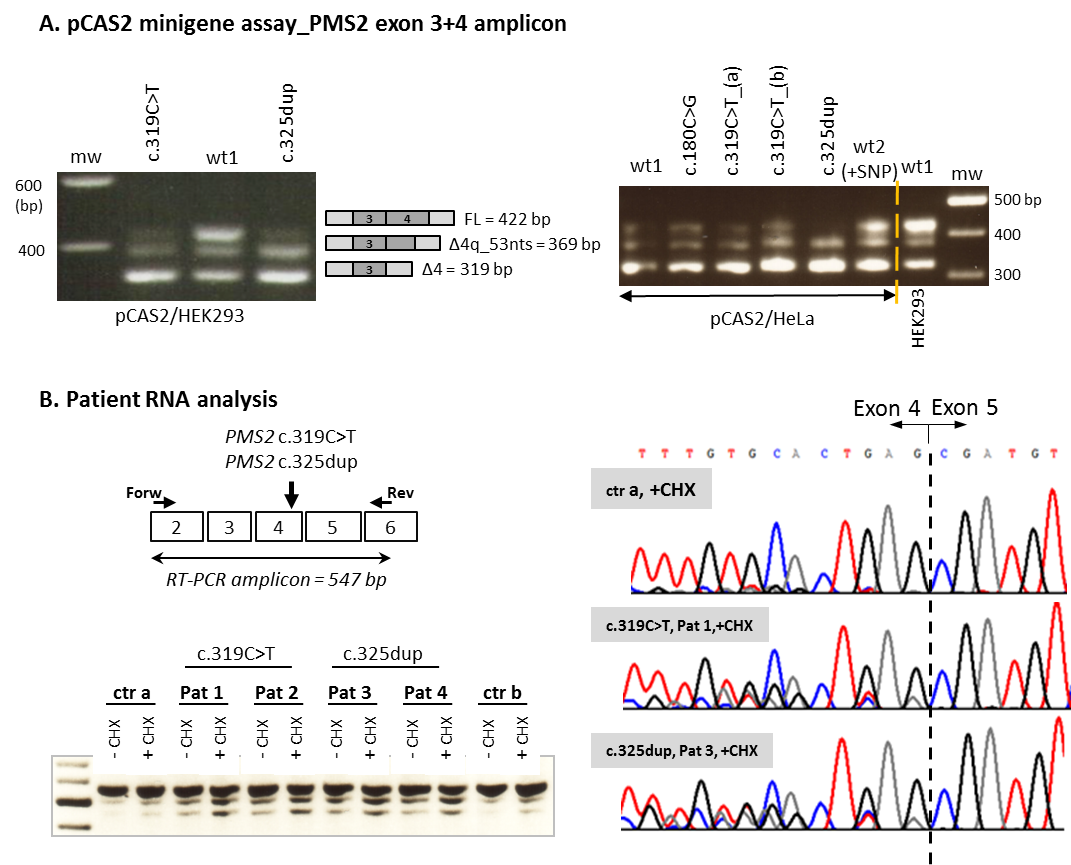
**

**Supp. Figure S1: Splicing reporter minigene assays (A) and patient RNA analyses (B) for *PMS2* c.319C>T and c.325dup. A:** Agarose gels showing splice patterns produced by pCAS2 minigene constructs transfected in HEK293 and HeLa cells. The wildtype 1 (wt1) allele from the *PMS2* exon 3+4 amplicon, derived from patient 1, carrier of c.319C>T, produce splice patterns with a major shift towards exon exclusion in HeLa compared to HEK293 cells. In wildtype 2 (wt2), this effect seems less pronounced (wt 2 only tested in HeLa). In wt2 the minor allele of the SNP c.288C>T (rs12532895) is present. This SNP is in trans with the c.325dup variant in the genomic DNA of patient 4. The agarose gel at the right shows RT-PCR products performed in one experiment on cDNA samples produced at different time points. Variant c.319C>T (a) and c319C>T (b) indicate two independent transfections with the same construct. PMS2 c.180C>G produces both in HeLa and in HEK293 the same splice pattern as wt1 (HEK293 transfection not shown for this variant). **B:** RT-PCR of PMS2 exon 2-6 on patient RNA derived from cultured lymphocytes with (+CHX) and without (-CHX) NMD inhibition. For each variant RNA samples from two related patients were tested. Sequence chromatograms from RT-PCR products (performed on RNA samples with NMD inhibition), sequenced with the Reverse primer in PMS2 exon 6, are shown as reverse complement sequence. Minor up-regulation of the alternative transcripts Δ4q_53nts and Δ4 is observed in RNA from patients carrying heterozygous *PMS2* c.319C>T and *PMS2* c.325dup, respectively. Alternative transcript expression is nearly absent in lymphocyte RNA from the two control individuals. **Abbreviations:** mw = molecular weight marker; wt = wildtype; FL = full-length transcript; bp = base pair; Δ =skip (Δx=skip of exon x); q = donor-site shift, followed by the number of nucleotides (nts) that are skipped.

**Comment on splicing results observed for variants in this genomic region**

Both minigene assays (pCAS2/HEK293) as patient RNA analyses show a shift towards alternative transcript expression associated with *PMS2* c.319C>T and c.325dup. The exact differences in the rate of expression of the FL and alternative transcripts cannot be deciphered from the agarose gel and sequence chromatograms. In the patient RNA the presence of the wildtype allele producing FL and alternative transcripts, also confounds the assessment of expression rate of the transcripts produced by the variant allele. A more quantitative approach should be applied to assess differences in expression between minigene assays and patient RNA analyses, and between control and patient RNA. Minigene assays performed in HeLa seem less reliable for this amplicon.


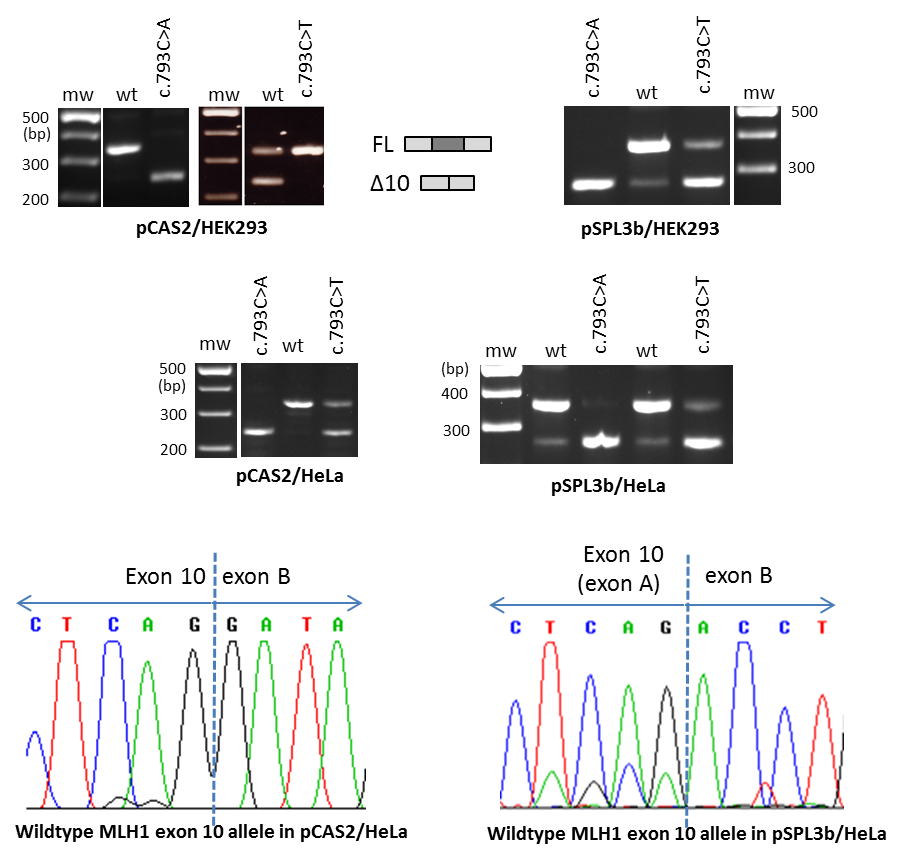


**Supp. Figure S2:** Minor quantitative differences in exon exclusion for the wildtype MLH1 exon 10 and the MLH1 c.793C>T constructs in the pCAS2 vector compared with pSPL3b. No differences between vectors and cell lines are observed for MLH1 c.793C>A. The wildtype allele shows a minor shift of FL transcript expression to exon 10 exclusion (Δ10) in the pSPL3b vector compared to pCAS2 on agarose gels and sequence chromatograms (chromatograms presented as reverse complement sequences, derived from RT-PCR products sequenced with the Reverse primer in exon B; only chromatograms of HeLa transfections are shown). **Abbreviations:** mw = molecular weight marker; wt = wildtype; FL = full-length transcript; bp = base pair


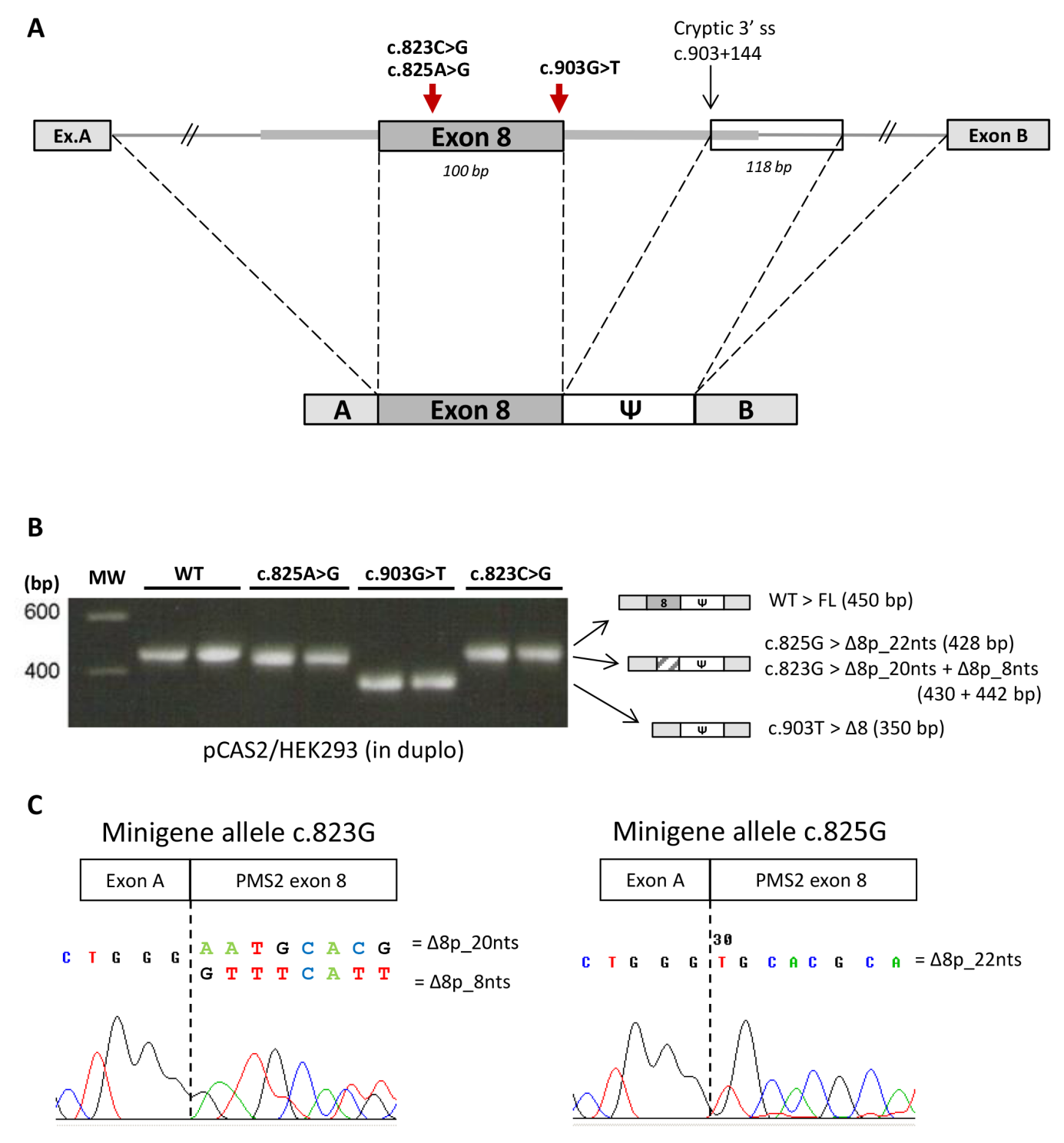


**Supp. Figure S3:** Artificial pseudo-exon inclusion in transcripts produced by *PMS2* exon 8 wildtype and variant alleles in pCAS2 transfected in HEK293 (in HeLa transfections the same splice patterns were observed, data not shown). **A:** Schematic representation of the *PMS2* exon 8 minigene design (above) and the FL transcript with artificial pseudo-exon (Ψ) inclusion (below). Red arrows indicate the position of the exonic variants tested in this study; the black arrow indicates the 3’cryptic splice site (ss) in *PMS2* intron 8 that is used a s acceptor in both pCAS2 as in pSPL3b minigene assays (size of pseudo-exon in pCAS2 is 118 bp, in pSPL3b 133 bp (pSPL3b data not shown). **B:** Minigene assays for *PMS2* c.825A>G, c.903G>T, and c.823C>G, with left the RT-PCR products on agarose gel, and right the schematic representations of the transcripts. Minigene constructs were transfected in duplo (at the same time point) in this experiment. **C.** Sequence chromatograms of RT-PCR products from pCAS2/HEK293 minigene assays for *PMS2* c. 823C>G (left) and c.825A>G (right). The variant constructs produced two (c.823G allele) and one (c.825G allele) aberrant transcripts, respectively. **Abbreviations:** MW = molecular weight marker; WT = wildtype; FL = full-length transcript; bp = base pair; Δ =skip (Δ8=skip of exon 8); p = acceptor-site shift, followed by the number of nucleotides (nts) that are skipped.

| **Supp. Table S1** Minigene amplicons: design and primer sequences^a^ | | | | | |
| --- | --- | --- | --- | --- | --- |
| **minigene amplicon** | **size (bp)** | **size (bp) of flanking intron sequences + [(pseudo)exon(s)]** | **Forward primer^b^** | **Reverse primer^b^** | **annealing T** |
| MLH1 exon 2 | 631 | 305-[91]-235 | [tail]-ATTAGTTGAGAAGAGACATC | [tail]-TCCCACCACTATAATGTAAG | 60 |
| MLH1 exon 3 | 474 | 115-[99]-260 | [tail]-AAAACTCTTCTAAGAGGATT | [tail]-ACAAGAAAACTTCATAGGTG | 51 |
| MLH1 exon 6 | 458 | 169-[92]-197 | [tail]-TTCTTCTGTTAATGCTGTCT | [tail]-TGATCTAAAGACCAACTGCT | 56 |
| MLH1 exon 10 | 594 | 246-[94]-254 | [tail]-GAGAATGTACTGCCTTATCA | [tail]-GCATGGCATTCCTTTTATTG | 58 |
| MLH1 exon 14 | 697 | 288-[109]-300 | [tail]-CTTTTAATAAAGTTGACCTC | [tail]-CTTCTTGGGTTATTTCTTAC | 51 |
| MLH1 exon 17 + 18 | 890 | 247-[93]-294-[114]-142 | [tail]-ATCTTGATGTGTTCTTTGGA | [tail]-TGAATAAAAGCTGACTGTTG | 55 |
| PMS2 exon 2 | 458 | 174-[140]-144 | [tail]-GATTGTAAAACAGTGTTTCT | [tail]-ACATAATAGGTGCTAACTTC | 58 |
| PMS2 exon 3 + 4 | 715 | 177-[87]-179-[103]-169 | [tail]-CTAGTAAATAGCCAGAAAGG | [tail]-GCTCACATTTCAGAAGTACTAT | 58 |
| PMS2 exon 6 | 484 | 208-[168]-98 | [tail]-ACATTGGGAAGTGAGAGGAC | [tail]-TTCTAAGATTTTATTCTCC | 58 |
| PMS2 exon 8 | 428 | 136-[100]-192 | [tail]-TGAACTGCAATAGTGATCCT | [tail]-AGTCTTCAGTTGAAACATCT | 58 |
| PMS2 exon 10 | 826 | 400-[156]-270 | [tail]-GTGAATGAAATAATAGATGG | [tail]-AAAACTAGAGGTACTTGGAG | 52 |
| PMS2 exon 12^c^ | 1507 | 468-[168]-871 | [tail]-AGAGTTGGATGAATGAATAA | [tail]-ATCTGCTATATGAATACTTA | 60 |
| PMS2 exon 14^c^ | 805 | 403-[170]-232 | [tail]-TGAGTTTCCTCTCTTTCAGG | [tail]-AGAATACTGCTTAACAGAGG | 58 |
| MSH2 exon 4 | 821 | 201-[147]-473 | [tail]-GAACGTAATTTTATATTTTG | [tail]-TCCAAGTACATAAACTATAA | 47 |
| MSH2 exon 13 | 482 | 138-[205]-139 | [tail]-TGTGGTTCTGCCTTTATATG | [tail]-AAAGTCCACAGGAAAACAAC | 55 |
| MSH6 exon 5 | 659 | 196-[266]-197 | [tail]-CTATCTTTTAGCTTTCCCTT | [tail]-TTCCCCTTAACATTAAGCAT | 53 |
|  |  |  |  |  |  |
| MSH2 intron 1 | 849 | 320-[75]-454 | [tail]-CTGGAAAAGCTGTAGACACT | [tail]-CAGATTTGAGATATTACATG | 57 |
| APC intron 4^d^ | 715 | 215-[167]-333 | [tail]-ATTATCAGGCACCATTGGCA | [tail]-GAGGTACTTCTTCAGAATGG | 56 |
| PKD2 intron 4 | 724 | 348-[54]-322 | [tail]-AAAATAAGTTCAGTGACTCT | [tail]-GAAGGTACAAGAAGAATATA | 58 |
| MSH2 intron 14 | 409 | 194-[71]-144 | [tail]-TCAGGAACTTTTAGTAGATC | [tail]-CACGTAAAGTGTCACTAATT | 55 |

**^a^**Minigene plasmids were sequenced for verification with the vector-specific sequence primers pCAS-seq-F: 5’- GGGGTCAATAGCAGTGAGAG-3’; pCAS-seq-R: 5’- GCTCCATTTCACAGGTAGAGA-3’; pSPL3b-seq-F: 5’- CTCCTTGGGATGTTGATGAT-3’; pSPL3b-seq-R: 5’- GCCCAAACATTCTGTACCTC-3’. RT-PCR analysis of the minigene transcripts was performed with vector-specific primers located in the vector exons A and B (pCAS-KOI-F: 5’- TGACGTCGCCGCCCATCAC-3’ and pCAS-2R: 5’- ATTGGTTGTTGAGTTGGTTGTC-3’ for pCAS2; SD6-Forw: 5’- TCTGAGTCACCTGGACAACC -3’ and SA2-Rev: 5’- ATCTCAGTGGTATTTGTGAGC-3’ for pSPL3b).

**^b^**Forward primers include a tail of 10 nts with restriction site BamHI for pCAS2 (AGTCGGATCC) and XhoI for pSPL3b (AGTCCTCGAG). Reverse primers carry a tail with restriction site MluI (AGCTACGCGT) for pCAS2 and EcoRV (AGCTGATATC) for pSPL3b. Restrictionsites are underlined.

**^c^***PMS2* primers in regions homologous to pseudogene sequences are designed on *PMS2*-specific nucleotides (or at least one of a primer pair) according to *PMS2* reference sequence NG_008466.1, accept for amplicon *PMS2* exon 12 whose primers anneal to both *PMS2* and *PMS2CL*. The forward primer for amplicon *PMS2* exon 14 is designed on a PSV (paralogous sequence variant) using the presumed *PMS2*-specific nucleotide as reported in the *PMS2* reference sequence.

**^d^**10 ul GC enhancer (Q5) was added to the PCR reaction (in a total volume of 50 ul).PCR was performed with Q5 polymerase.

| **Supp. Table S2** Primers used for site-directed mutagenesis (SDM) in pCAS2 wildtype constructs | | | | |
| --- | --- | --- | --- | --- |
| **Variant** | **minigene amplicon for wildtype construct** | **SDM_Forward primer** | **SDM_Reverse primer** | **annealing T** |
| PMS2 c.538-3C>G | PMS2 exon 6 | GTTTTTTAAAgAGGAGTATGCCAAAATG | ACAAACACAATATTCTACATTAC | 59 |
| PMS2 c.989-1G>T | PMS2 exon 10 | TTTTGCCTTAtAATGCGTTGATATC | AAGAAAACATATTTATTATGTTTAAATTCAC | 60 |
| PMS2 c.989-2A>G | PMS2 exon 10 | TTTTTGCCTTgGAATGCGTTG | AGAAAACATATTTATTATGTTTAAATTCAC | 59 |
| MSH2 c.212-478T>G | MSH2 intron 1 | CTGAAATGAGgTAAGGCTTTG | CATTAATTCAAAATTCCAGTCC | 58 |
| APC c.532-941G>A | APC intron 4 | AAAGGGAAAAaGGTGAGAACTAATC | GCAACATACAGATGACATTC | 59 |
| PKD2 c.1094+507A>G | PKD2 intron 4 | GTACACGTGCgTATTTACAGG | AAATATATACCTAAAATCTCAAACATTTG | 58 |

**Suppl table S3a** Specification of minigene vectors and transfection cell lines used in splicing assays for each variant tested in this study. Minor alleles of SNPs present in the variant construct *in cis* with the variant of interest are recorded. Twelve of the 36 variant constructs were used in a second or third set of transfection experiments; no difference in splice patterns was observed between any of these independent experiments.

|  |  |  |  |  | number of independent transfections | | | |
| --- | --- | --- | --- | --- | --- | --- | --- | --- |
|  |  |  |  |  | pCAS2 | | pSPL3b | |
| **Gene** | **minigene amplicon** | **Variant tested in this study** | **DNA source for minigene** | **other variants in cis with variant of interest in minigene constructs** | **HEK293** | **HeLa** | **HEK293** | **HeLa** |
| MLH1 | 2 | c.122A>G | patient DNA | none | 1 | 1 |  |  |
| MLH1 | 3 | c.277A>G | patient DNA | none | 1 |  |  |  |
| MLH1 | 6 | c.543C>G | patient DNA | c.454-125A>C (not in dbSNP) | 2 |  |  |  |
| MLH1 | 6 | c.545G>A | patient DNA | none | 1 | 1 |  |  |
| MLH1 | 10 | c.791-1G>C | patient DNA | none | 1 | 2 |  |  |
| MLH1 | 10 | c.793C>A | patient DNA | none | 1 | 1 | 1 | 1 |
| MLH1 | 10 | c.793C>T | patient DNA | none | 2 | 3 | 1 | 1 |
| MLH1 | 10 | c.882C>T | patient DNA | none | 1 | 1 |  |  |
| MLH1 | 10 | c.883A>G | patient DNA | none | 2 | 2 |  |  |
| MLH1 | 14 | c.1633A>G | patient DNA | none | 1 |  |  |  |
| MLH1 | 14 | c.1667+1del | patient DNA | none | 1 |  |  |  |
| MLH1 | 17 + 18 | c.2103G>A | patient DNA | c.1990-121C>T (rs2241031) | 1 |  |  |  |
| PMS2 | 2 | c.139C>T | patient DNA | c.59G>A, p.R20Q (rs10254120) | 1 | 1 |  |  |
| PMS2 | 2 | c.163+2T>C | patient DNA | none | 1 | 1 |  |  |
| PMS2 | 3 + 4 | c.180C>G | patient DNA | none | 2 | 2 |  |  |
| PMS2 | 3 + 4 | c.319C>T | patient DNA | none | 3 | 2 |  |  |
| PMS2 | 3 + 4 | c.325dup | patient DNA | none | 2 | 2 |  |  |
| PMS2 | 6 | c.538-3C>G | SDM | none | 1 | 1 |  |  |
| PMS2 | 6 | c.614A>C | patient DNA | c.705+17A>G; rs62456182 |  | 1 |  |  |
| PMS2 | 6 | c.687T>C | patient DNA | c.705+17A>G; rs62456182 |  | 1 |  |  |
| PMS2 | 8 | c.823C>G | patient DNA | none | 2 | 1 | 1 |  |
| PMS2 | 8 | c.825A>G | patient DNA | c.903+84C>T (rs3815383); c.903+100T>G (rs12534423) | 2 | 1 | 1 |  |
| PMS2 | 8 | c.903G>T | patient DNA | none | 2 | 1 |  |  |
| PMS2 | 10 | c.989-1G>T | SDM | none | 1 | 1 |  |  |
| PMS2 | 10 | c.989-2A>G | SDM | none | 1 | 1 |  |  |
| PMS2 | 10 | c1144+2T>A | patient DNA | none | 1 | 1 |  |  |
| PMS2 | 12 | c.2174+1G>A | patient DNA | c.2007-151A>G (rs2711201); c.2007-75del (rs34745403); c.2174+277C>G (rs58032887) | 1 | 2 |  |  |
| PMS2 | 14 | c.2445+1G>T | patient DNA | none | 1 | 1 |  |  |
| MSH2 | 4 | c.728G>A | patient DNA | none | 1 |  |  |  |
| MSH2 | 13 | c.2006G>T | patient DNA | none | 1 |  |  |  |
| MSH6 | 5 | c.3438+1G>A | patient DNA | c.3173-101C>G (rs2072447) | 1 |  |  |  |
| MSH2 | i01 | c.212-478T>G | SDM | c.212-405_212-404delATinsGG (rs34057196) | 1 |  |  |  |
| APC | i05 | c.532-941G>A | SDM | none | 2 | 1 |  |  |
| APC | i05 | c.532-941G>A | patient DNA | c.532-845A>G (rs77939389) | 1 | 1 |  |  |
| PKD2 | i04 | c.1094+507A>G | SDM | rs2725220 (G allele); rs2725218 (G allele); rs7696304 (A allele) | 1 |  |  |  |
| MSH2 | i14 | c.2459-834A>G | patient DNA | none | 1 | 1 |  |  |

**Suppl table S3b** Minor alleles of SNPs present in the wildtype constructs used in this study. For two amplicons wildtype constructs available from two different DNA sources harboured either minor SNP alleles or major (=reference) SNP alleles (indicated as “none”); these SNPs however did not influence splicing in the minigene assays.

| **Gene** | **minigene amplicon** | **DNA source for minigene** | **wildtype minigenes with SNP minor allele** |
| --- | --- | --- | --- |
| MLH1 | 2 | patient DNA | none |
| MLH1 | 3 | patient DNA | none |
| MLH1 | 6 | patient DNA | none |
| MLH1 | 10 | patient DNA | none |
| MLH1 | 14 | patient DNA | none |
| MLH1 | 17 + 18 | patient DNA | none |
| PMS2 | 2 | patient DNA | none |
| PMS2 | 3 + 4 | patient DNA | 2 diff wt minigenes: none / c.288C>T (rs12532895) |
| PMS2 | 6 | SDM | none |
| PMS2 | 8 | patient DNA | none |
| PMS2 | 10 | SDM | none |
| PMS2 | 10 | patient DNA | none |
| PMS2 | 12 | patient DNA | 2 diff wt minigenes; plasmid 1: none; plasmid 2 : c.2007-151A>G (rs2711201); c.2007-75del (rs34745403); c.2174+61A>G (rs2692554); c.2174+103C>T (rs2692553) |
| PMS2 | 14 | patient DNA | none |
| MSH2 | 4 | patient DNA | none |
| MSH2 | 13 | patient DNA | none |
| MSH6 | 5 | patient DNA | none |
| MSH2 | i01 | SDM | c.212-405_212-404delATinsGG (rs34057196) |
| APC | i05 | SDM | none |
| APC | i05 | patient DNA | none |
| PKD2 | i04 | SDM | rs2725220 (G allele); rs2725218 (G allele); rs7696304 (A allele) |
| MSH2 | i14 | patient DNA | none |

| Supp. Table S4 Information on patient RNA analysis (RT-PCR) of the 35 variants described in this study | | | |  |
| --- | --- | --- | --- | --- |
| variant (predicted protein change)^a^ | **source of transcript description^b^** | **source of mRNA isolated from the patient^c^** | **description of splice affect in patients mRNA^d^** | **RNA effect (in patients mRNA)^a^** |
| MLH1 c.122A>G (p.Asp41Gly) | LUMC; MUMC | STCLs + NMD inhibition | no aberrant splicing (FL + weak Δ2p_5nts, same as in controls); conflicts with Sharp, 2004 | r.122a>g |
| MLH1 c.277A>G (p.Ser93Gly) | *no patient RNA available* | *no patient RNA available* | *no patient RNA available* | r.(277a>g) |
| MLH1 c.543C>G (p.=) | LUMC | STCLs + NMD inhibition | Δ6; no FL from variant allele | r.454_545del |
| MLH1 c.545G>A (p.Arg182Lys) | LOVD microattribution ORCID Mensenkamp&Ligtenberg | unknown | Two aberrant transcripts: Δ6 + Δ6q_4nts, no FL from variant allele | r.[454_545del, 542_545del] (*as in LOVD)* |
| MLH1 c.791-1G>C^e^ | Thompson, 2013 | LCLs + NMD inhibition | upregulation of alternative transcript Δ10; absence of FL transcript from variant allele not assessed | r.791_884del (*as in LOVD)* |
| MLH1 c.793C>A (p.Arg265Ser)^e^ | LUMC | STCLs without NMD inhibition (RNA from STCLs with NMD inhibition was of poor quality, RIN 3.8, causing an unreliable transcript profile) | Δ10 (low expression because of NMD)); no FL from variant allele; | r.791_884del (*as in LOVD)* |
| MLH1 c.793C>T (p.Arg265Cys)^e^ | Casey, 2005; Yuen, 2002; LOVD microattributions ORCID:Leung et al, ORCID:Andreas Laner | Casey et al 2005: RNA (no NMD inhibition) from hybrid cell lines, conversion technology; Yuen, 2002, and both microattributions source unknown | Casey, 2005: Δ(9+10)(in-frame) low expression of variant allele. Yuen, 2002: Δ10. LOVD microattributions report no FL expressed from variant allele | r.791_884del (*as in LOVD)* |
| MLH1 c.882C>T (p.=)^e^ | Auclair 2006, Spaepen 2006 | LCLs + NMD inhibition | Δ10; proof for absence of FL transcript from variant allele not shown in references | r.791_884del (*as in LOVD)* |
| MLH1 c.883A>G (p.Ser295Gly)^e^ | Casey, 2005; Goldschmidt, 2005 | Casey et al 2005: RNA (no NMD inhibition) from hybrid cell lines, conversion technology; Goldschmidt, 2005: PBLs | Casey, 2005: Δ(9+10); Goldschmidt, 2005: no FL transcript from variant allele | r.678_884del (*as in LOVD)*^e^ |
| MLH1 c.1633A>G (p.Thr545Ala) | LUMC | STCLs + NMD inhibition | no aberrant splicing | r.1633a>g |
| MLH1 c.1667+1delG | LUMC | STCLs + NMD inhibition | in-frame inclusion of 87 bp (c.1667+2_1667+88), no PTC created; absence of FL from variant allele not assessed | r.[1667+1del; 1667_1668ins1667+2_1667+88] |
| MLH1 c.2103G>A (p.=) | LUMC | STCLs + NMD inhibition | Δ18 (major) + Δ[17+18] (very minor). Both exon skips are in-frame. Minor, nearly visible FL expression from variant allele | r.[1990_2103del, 1897_2103del] |
| PMS2 c.139C>T (p.=) | LUMC | STCLs + NMD inhibition | no aberrant splicing | r.139c>u |
| PMS2 c.163+2T>C | LUMC | STCLs + NMD inhibition | Δ2 (out of frame; no NMD); absence of FL from variant allele not assessed | r.24_163del (*as in LOVD)* |
| PMS2 c.180C>G (p.Asp60Glu) | *no patient RNA available* | *no patient RNA available* | *no patient RNA available* | r.(180c>g) |
| *(Suppl Table S4 continued)* | |  |  |  |
| variant (predicted protein change)^a^ | **source of transcript description^b^** | **source of mRNA isolated from the patient^c^** | **description of splice affect in patients mRNA^d^** | **RNA effect (in patients mRNA)^a^** |
| PMS2 c.319C>T (p.Arg107Trp) | LUMC | STCLs + NMD inhibition | upregulation of alt.transcripts Δ4 + Δ4q_53nts (OOF, subject to NMD); minor FL expression from variant allele (missense) | r.[=, 301_353del, 251_353del] |
| PMS2 c.325dupG | LUMC | STCLs + NMD inhibition | upregulation of alt.transcripts Δ4 + Δ4q_53nts; minor FL from variant allele (but subject to NMD caused by PTC) | r.[325dup, 301_353del, 251_353del] |
| PMS2 c.538-3C>G | Borras, 2013 | STCLs + NMD inhibition | two aberrant transcripts: skip of exon 6 (in-frame) and Δ6p_49nts; absence of FL from variant allele not assessed | r.[538_586del, 538_705del] (*as in LOVD)* |
| PMS2 c.614A>C (p.Gln205Pro) | LUMC | STCLs + NMD inhibition | no aberrant splicing (same as in contols: FL + weak Δ6p_49nts) | r.614a>c |
| PMS2 c.687T>C (p.=) | LUMC | STCLs + NMD inhibition | no aberrant splicing (same as in contols: FL + weak Δ6p_49nts) | r.687u>c |
| PMS2 c.823C>G (p.Gln275Glu) | *no patient RNA available* | *no patient RNA available* | *no patient RNA available* | r.(spl?) |
| PMS2 c.825A>G (p.=) | LUMC | STCLs + NMD inhibition | Δ8p_22nts; no FL transcript from variant allele | r.804_825del |
| PMS2 c.903G>T (p.Lys301Asn) | LOVD microattribution ORCID Mensenkamp&Ligtenberg | unknown | Δ8; absence of FL from variant allele not reported | r.804_903del (*as in LOVD)* |
| PMS2 c.989-2A>G | Borras, 2013 | STCLs + NMD inhibition | skip of exon 10 (in-frame); absence of FL from variant allele not assessed | r.989_1144del (*as in LOVD)* |
| PMS2 c.989-1G>T | Sjursen, 2009 | PAXgene blood RNA | two aberrant transcripts: Δ10 and Δ10p_27nts (both in-frame); no FL transcript in homozygous patient | r.989_1144del,r. 989_1015del (*as in LOVD)* |
| PMS2 c.1144+2T>A | LUMC | STCLs + NMD inhibition | Δ10; absence of FL from variant allele not assessed | r.989_1144del (*as in LOVD)* |
| PMS2 c.2174+1G>A | LUMC | STCLs + NMD inhibition | three aberrant transcripts visible on agarose, but sequence deciphered for only two: Δ12 + ▼12q_421nts. No FL from variant allele (tested with coding SNP p.P470S); partial NMD observed (Δ12 is in frame, not causing NMD) | r.[2007_2174del, 2174+1g>a; 2174_2175ins2174+1_2174+421, 2174_2175ins?] |
| PMS2 c.2445+1G>T | LUMC | STCLs + NMD inhibition | one aberrant transcript: ▼14q_85nts, creates PTC; absence of FL from variant allele not assessed | r.[2445+1g>u; 2445_2446ins2445+1_2445+85] |
| MSH2 c.728G>A (p.Arg243Gln) | LUMC | STCLs + NMD inhibition | no aberrant splicing | r.728g>a (*as in LOVD)* |
| MSH2 c.2006G>T (p.Gly669Val) | LUMC | STCLs + NMD inhibition | Δ13; no FL transcript produced by variant allele | r.2006_2210del (*as in LOVD)* |
| MSH6 c.3438+1G>A | *no patient RNA available* | *no patient RNA available* | *no patient RNA available* | r.(3173_3438del) (*as in LOVD)* |
| MSH2 c.212-478T>G | Clendenning, 2011 | LCLs + NMD inhibition | introduction of 75 bp in mRNA (c.212-553_c.212-479) creating a stopcodon, and predicted to result in a truncated protein of 94 AA | r.211_212ins212-553_212-479 (*as in LOVD)* |
| APC c.532-941G>A | Spier, 2012 | PAXgene blood RNA | insertion of 167 bp (r.531_532ins532-1106_532-940) | r.531_532ins532-1106_532-940 (*as in LOVD)* |
| PKD2 c.1094+507A>G | Rossetti, 2012 | LCLs | no aberrant transcription observed | r.(?) |
| MSH2 c.2459-834A>G | *no patient RNA available* | *no patient RNA available* | *no patient RNA available* | r.(?) |

**^a^** Nomenclature according to HGVS guidelines. The following reference sequences are used: NM_000249.3 for *MLH*1, NM_000535.5 for *PMS2*, NM_000251.2 for *MSH2*, NM_000179.2 for *MSH6*, NM_000038.5 for *APC*, and NM_000297.2 for *PKD2*.

**^b^** Transcript descriptions are derived from patient RNA analyses performed at our lab (LUMC=Leiden University Medical Centre; research section Human Genetics department; The Netherlands), reported at LOVD (<http://chromium.liacs.nl/LOVD2/colon_cancer/home.php>), or reported in literature (Auclair et al., 2006, Hum Mutat 27:145; Borras et al., 2013, J Med Genet 50:552; Casey et al., 2005, JAMA 293:799; Clendenning et al, 2011., Fam Cancer 10:297; Goldschmidt et al., 2005, Int J Cancer 116:808; Rossetti et al, 2012., J Am Soc Nephrol 23:915; Sjursen et al., 2009, Fam Cancer 8:179; Spaepen et al., 2006, Fam Cancer 5:179; Spier et al., 2012, Hum Mutat 33:1045; Thompson et al., 2013, Hum Mutat 34:200; Yuen et al., 2002, Oncogene 21:7585). MUMC = Maastricht University Medical Centre, The Netherlands

**^c^** LCLs = lymphoblastoid cell lines; NMD = nonsense-mediated decay; PBLs = peripheral blood lumphocytes; STCLs = short-term cultured lymphocytes

**^d^** Δ =skip (Δx=skip of exon x); ▼= inclusion of intronic sequence in transcript; p = acceptor-site shift, q = donor-site shift, p and q followed by the number of nts that are skipped or included; bp = base pair, nts = nicleotides, FL = full-length (reference) transcript, NMD = nonsense-mediated decay, OOF = out of frame, PTC = premature termination codon

**^e^** Patient RNA analyses of variants in or near *MLH1* exon 10 are hampered by the presence of many alternative transcripts (Δ9/10, Δ9_11, Δ10, Δ10/11; Thompson et al., Clin Genet. 2014 Jul 2. doi: 10.1111/cge.12450) that together with the FL transcript from the wildtype allele often hinder the exact characterization of the aberrant splicing profile produced by the mutant allele.When RNA without NMD inhibition is used, it is possible that only the in-frame Δ9/10 is reported as aberrant up-regulation caused by the variant while the other aberrant transcripts, degraded by NMD, are missed (as probably is the case for MLH1 c.883A>G by Casey et al., 2005).

**Supp. Table S5** Primer sequences and PCR conditions used for RT-PCR in the patient RNA analyses performed in our lab (LUMC; research section). Internal sequence primers are available on request.

| **RT-PCR amplicon** | **size (bp)** | **Forward primer** | **Reverse primer** | **Annealing T (x), extension time (y), and PCR conditions (A or B)^a^** | **variants analysed with this amplicon** |
| --- | --- | --- | --- | --- | --- |
| RT-MLH1-exon 1-19 | 2415 | ATGTCGTTCGTGGCAGGG | GGAATACTATCAGAAGGCAAG | 58^o^C, 2 min, A | MLH1 c.122A>G; c.543C>G; c.1633A>G; c.1667+1del; c.2103G>A |
| RT-MLH1 exon 1-5 | 411 | ATGTCGTTCGTGGCAGGG | GGCTTTCAGTTTTCCATCTG | 58^o^C, 45 sec, B | MLH1 c.122A>G |
| RT-MLH1 exon 8-14 | 1005 | AGGAGAGACAGTAGCTGATG | CCCACGAAGGAGTGGTTATG | 52^o^C, 1 min, B | MLH1 c.793C>A |
| RT-MLH1 exon 13-19 | 997 | TCGGGAAGATTCTGATGTGG | GGAATACTATCAGAAGGCAAG | 60^o^C, 1 min, B | MLH1 c.2103G>A |
| RT-PMS2 exon 1-11 | 1338 | AACTTTCCCAGTCCCCGAGG | CTTGTTCTCTGTTGTGTGAC | 58^o^C, 90 sec, A | PMS2 c.139C>T; c.319C>T; c.325dup; c.614A>C; c.687T>C; c.825A>G; c.1144+2T>A |
| RT-PMS2 exon 10-15 | 1648 | GTGATGTCAACAAGCTAAATG | GAAAAGGTTCTCAAGATCAC | 58^o^C, 90 sec, A | PMS2 c.2174+1G>A; c.2445+1G>T |
| RT-PMS2 exon 2-6 | 547 | TACAGAACCTGCTAAGGCCA | TGCATGTAAGACCTGGACCA | 58^o^C, 45 sec, B | PMS2 c.319C>T; c.325dup |
| RT-PMS2 exon 10-12/13 border (wt transcript- specific) | 1067 | AGCCACTGCTGGATGTTGAA | GTTGAGAGTCTGAGGTGCTA | 58^o^C, 70 sec, A | PMS2 c.2174+1G>A [no wildtype transcript from mutated allele, using SNP p.P470S in exon 11 ) |
| RT-MSH2 exon 1-10 | 1586 | CTTAGTGGGTGTGGGGTCGCGCATT | CTGTTTGCCAGGGTCCAAGC | 63^o^C, 90 sec, A | MSH2 c.728G>A |
| RT-MSH2 exon 6-16 | 1755 | ATGGATAAGAACAGAATAGAGGAG | TCACGTAGTAACTTTTATTCGTG | 58^o^C, 90 sec, A | MSH2 c.2006G>T |

^a^RT-PCR was performed using either (A) the Expand^TM^ Long Template PCR system (Roche Diagnostics) in combination with buffer 3 or (B) FastStart Taq DNA Polymerase (Roche Diagnostics) with buffer Green. 1 ul cDNA was used as template in a total reaction volume of 25 ul. PCRs were performed on a PTC-200 DNA Engine Cycler (Biorad) using the following cycling programs for(A) 94 ^o^C for 2 minutes (min); ten cycles of 94 ^o^C for 20 seconds (sec), x^o^C for 30 sec, 68 ^o^C for y sec; 24 cycles of 94 ^o^C for 20 seconds (sec), x ^o^C for 30 sec, 68 ^o^C for y sec + 20 sec added in each cycle; finishing with 68 ^o^C for 7 minutes; and for (B) 95 ^o^C for 8 min; 38 cycles of 95 ^o^C for 30 sec, x^o^C for 30 sec, 72 ^o^C for y sec; finishing with 72 ^o^C for 5 minutes.
